# The effects of TGF-β-induced activation and starvation of vitamin A and palmitic acid on human stem cell-derived hepatic stellate cells

**DOI:** 10.1186/s13287-024-03852-8

**Published:** 2024-07-23

**Authors:** Ingrid Wilhelmsen, Thomas Combriat, Andrea Dalmao-Fernandez, Justyna Stokowiec, Chencheng Wang, Petter Angell Olsen, Jonas Aakre Wik, Yuliia Boichuk, Aleksandra Aizenshtadt, Stefan Krauss

**Affiliations:** 1 Department of Immunology and Transfusion Medicine, Oslo University Hospital, P.O. Box 4950, Nydalen, Oslo, 0424 Norway; 2 Hybrid Technology Hub - Centre of Excellence, Institute of Basic Medical Sciences, University of Oslo, P.O. Box 1110, Blindern, Oslo, 0317 Norway; 3 Section for Pharmacology and Pharmaceutical Biosciences, Department of Pharmacy, University of Oslo, P.O. Box 1068, Blindern, Oslo, 0316 Norway; 4 Department of Transplantation Medicine, Institute for Surgical Research, Oslo University Hospital, P.O. Box 4950, Nydalen, Oslo, 0424 Norway

**Keywords:** Hepatic stellate cells (HSC), Human pluripotent stem cells (hPSC), Fibrosis modeling, In vitro, Vitamin A, Lipid droplets, Energy metabolism, TGF-β, Retinol, Palmitic acid

## Abstract

**Background:**

Hepatic stellate cells (HSC) have numerous critical roles in liver function and homeostasis, while they are also known for their importance during liver injury and fibrosis. There is therefore a need for relevant in vitro human HSC models to fill current knowledge gaps. In particular, the roles of vitamin A (VA), lipid droplets (LDs), and energy metabolism in human HSC activation are poorly understood.

**Methods:**

In this study, human pluripotent stem cell-derived HSCs (scHSCs), benchmarked to human primary HSC, were exposed to 48-hour starvation of retinol (ROL) and palmitic acid (PA) in the presence or absence of the potent HSC activator TGF-β. The interventions were studied by an extensive set of phenotypic and functional analyses, including transcriptomic analysis, measurement of activation-related proteins and cytokines, VA- and LD storage, and cell energy metabolism.

**Results:**

The results show that though the starvation of ROL and PA alone did not induce scHSC activation, the starvation amplified the TGF-β-induced activation-related transcriptome. However, TGF-β-induced activation alone did not lead to a reduction in VA or LD stores. Additionally, reduced glycolysis and increased mitochondrial fission were observed in response to TGF-β.

**Conclusions:**

scHSCs are robust models for activation studies. The loss of VA and LDs is not sufficient for scHSC activation in vitro, but may amplify the TGF-β-induced activation response. Collectively, our work provides an extensive framework for studying human HSCs in healthy and diseased conditions.

**Supplementary Information:**

The online version contains supplementary material available at 10.1186/s13287-024-03852-8.

## Background

Hepatic stellate cells (HSCs) are liver non-parenchymal cells that are crucial for extracellular matrix (ECM) homeostasis and vitamin A (VA) storage. HSCs reside in the perisinusoidal space of Disse and exhibit a quiescent phenotype in homeostatic conditions while they can be induced to an activated phenotype during liver injury. One of the prominent features of quiescent HSCs is the presence of cytoplasmic lipid droplets (LDs) specialized for VA storage, mainly in the form of retinyl esters [1]. Activated HSCs, on the other hand, have transdifferentiated into proliferative, pro-inflammatory myofibroblast-like cells with enhanced ECM production and altered metabolism [2, 3]. During chronic liver disease, activated HSCs will continuously deposit excessive ECM to the perisinusoidal space of Disse, comprising a key feature in fibrotic scarring that may eventually lead to hepatic cirrhosis [4, 5].

The loss of the VA-containing LDs is widely reviewed as a hallmark of HSC activation [1, 6]. This phenomenon has been extensively studied in rodent HSC models, where decreased VA levels correlate with HSC activation-related parameters in vivo [7, 8] and in vitro [9–11]. The same observation has been made for the loss of LDs in rodent HSC activation, here, too, both in vivo [12] and in vitro [13]. Similarly, reduced VA levels have been reported from diseased human liver biopsies [14] and human liver slices after activation by prolonged in vitro cultivation [15]. Despite this overwhelming consensus, the roles of VA and LDs in the HSC activation process are poorly understood.

Additionally, changes in VA and LD content and composition during HSC activation have been linked to alterations in cell energy metabolism in vitro, as in vivo studies of HSC energy metabolism are lacking [3, 16]. As HSC activation leads to an increased demand for protein production, in vitro studies on mouse HSCs have linked activation-related LD catabolism to increased mitochondrial β-oxidation [12] and mitochondrial oxidative phosphorylation (OxPhos) [17, 18]. Similarly, in vitro studies on culture-activated rodent HSCs and the human LX-2 cell line have shown increased glucose uptake, expression of glycolysis-related genes during early activation, and oxygen consumption rate, suggesting enhanced aerobic glycolysis as an activation characteristic [18–20].

However, results on VA and LDs in the HSC activation process vary based on 1) model species, 2) activation modality, and 3) methods of VA- and LD detection. Significant variation in VA storage exists between species [21–24], with differences in in vitro drug responses [25] and esterification enzymes [26] noted between mice, rats, and human LX-2 cell cultures. Furthermore, activation of HSCs showed varied transcriptomic profiles between in vivo-activated human primary HSCs (pHSCs) and those activated via prolonged in vitro culture, indicating the latter may not fully represent in vivo conditions [27]. Different activation modalities also produced contradicting results in rodent in vitro HSC studies, as research on mitochondrial metabolism showed that short-term activation induced by TGF-β, a potent HSC activator [28], led to increased mitochondrial fission in mouse HSCs [17] while prolonged culture-activation led to increased mitochondrial fusion in rat HSCs [18]. Lastly, a study comparing Oil Red O staining - a widely used method for LD detection - with protein immunofluorescence (IF) and electron microscopy (EM) for LD detection in rat HSCs surprisingly found that in contrast to the Oil Red O staining, IF and EM results showed a retention of LDs during activation [29]. As a whole, these insights make it problematic to generalize and translate the conclusions of animal HSC models to human HSCs.

Consequently, there is a need for predictive human HSC in vitro models, faithful methods of HSC activation, and reliable techniques for VA- and LD detection. Research using human pHSCs is challenging due to low donor availability, high donor variability, and rapid activation of the isolated HSCs during in vitro cultivation [27]. Similarly, immortalized human HSC cell lines, including LX-2, exhibit phenotypes comparable to that of activated human HSCs [30, 31]. To study the human HSC activation process in a more reproducible system, protocols for the generation of quiescent-like human pluripotent stem cell- (hPSC-) derived HSCs (scHSCs) have been developed [32–36]. We have previously characterized in vitro activation of 2D cultured scHSCs using the potent HSC activator TGF-β, demonstrating the suitability of human scHSCs for studying the short-term effects of HSC activation [37].

In this study, we employed human scHSCs derived from four different hPSC lines for testing the effect of TGF-β in the presence or absence of retinol (ROL) and palmitic acid (PA) on the transcriptome, VA, LDs, and cell energy metabolism in vitro. We chose to investigate the combinational effects of both ROL and PA starvation similar to previous in vitro studies on human pHSCs [38, 39] as ROL is a lipid-soluble compound [40]. For a comprehensive analysis of the approach, we used a wide variety of methods, including gene expression analysis by mRNA sequencing, UV autofluorescence imaging, holotomographic (HT) imaging using a Tomocube HT-X1 (Tomocube), confocal Raman spectroscopy, substrate oxidation assays, and protein analysis by immunofluorescence (IF) imaging and enzyme-linked immunosorbent assay (ELISA). Using this strategy we created an extensive human scHSCs framework for future human in vitro models of liver fibrosis development.

## Methods

### Culturing and maintenance of stem cells

hPSCs were cultured in E8 media (Thermo Fisher Scientific) on plates coated with 1% (v/v) Geltrex (Thermo Fisher Scientific). The hPSCs were incubated in a humidified 37 °C, 5% CO_2_ incubator, and the media was replenished every 24 h. Passaging was performed with 5 µM EDTA (Thermo Fisher Scientific) in DPBS (Thermo Fisher Scientific) and the hPSCs were replated as small colonies at dilutions from 1:3 − 1:5. Quality control of the hPSCs was regularly performed by IF imaging, RT-qPCR and flow cytometry for selected pluripotency markers. All cultured cells were checked for mycoplasma (BioNordica).

### scHSC differentiation

**Fig. 1:**
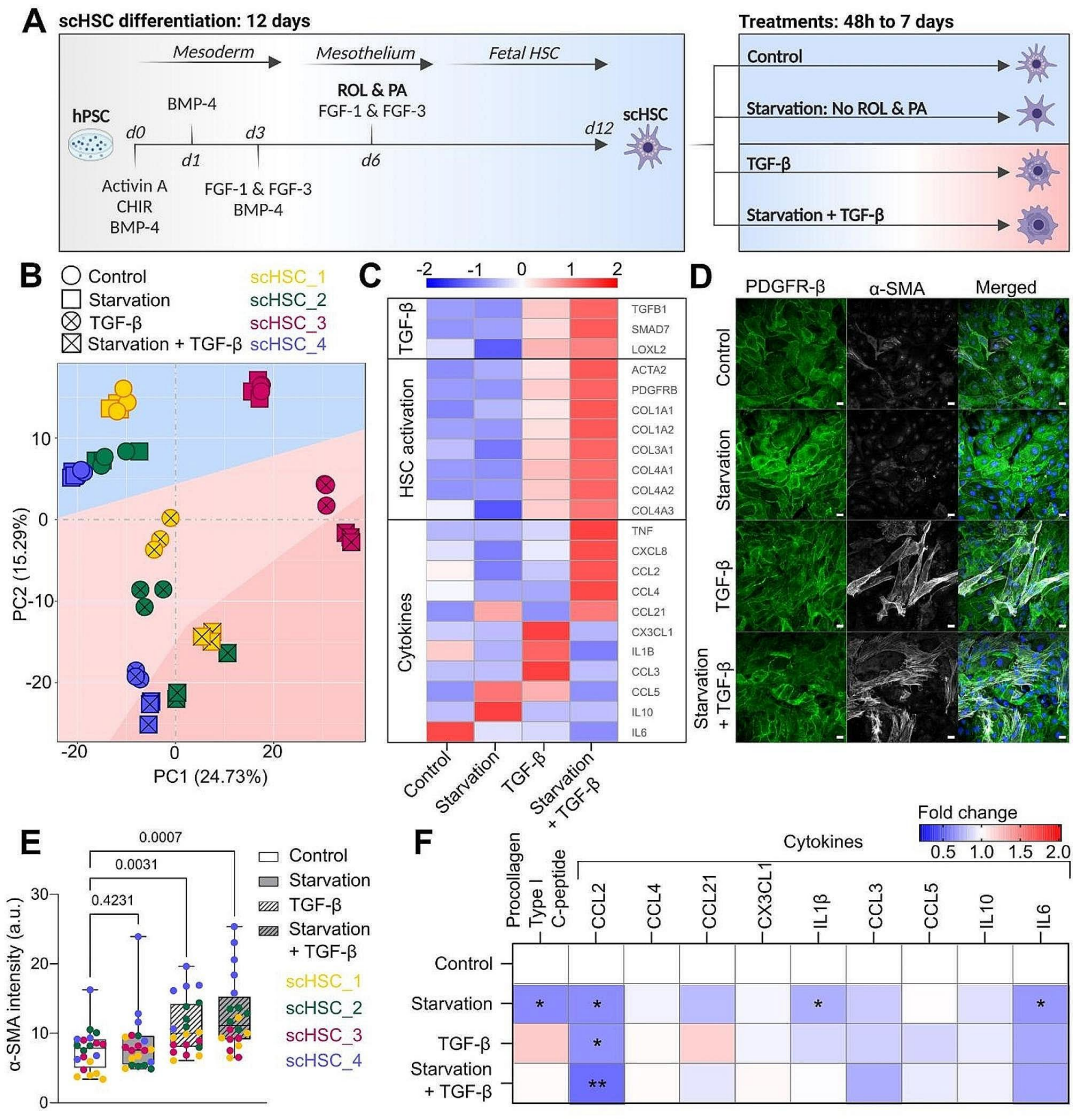
Retinol (ROL) and palmitic acid (PA) starvation and TGF-β-induced activation of scHSCs. (**A**) Schematic representation of the scHSC differentiation protocol and the subsequent experimental groups. ROL: Retinol, PA: Palmitic acid. (**B**) Principal component analysis (PCA) of mRNA sequencing data of scHSCs. *n* = 4 cell lines, with *N* = 3 technical replicates. (**C**) Heatmap showing the z-score of selected HSC activation-related genes from mRNA sequencing data of scHSCs. *n* = 4 cell lines, with *N* = 3 technical replicates. (**D**) Representative confocal immunofluorescence (IF) images of PDGFR-β and α-SMA in scHSC_4 after 48 h of treatment. Scale bars: 40 μm. (**E**) Quantification of the average integral α-SMA intensity within PDGFR-β positive areas of IF confocal images of scHSCs. *n* = 4 cell lines, *N* = 5 independent fields imaged. (**F**) Secretion levels of procollagen type I C-peptide and cytokines produced by scHSCs after 48 h of incubation, displayed as the fold difference compared to “Control” (statistical significance was calculated on the concentration values). *n* = 4 cell lines, *N* = 4 technical replicates. *p-value ≤ 0.05, **p-value ≤ 0.01. Statistical tests: Unpaired t-test with Welch’s correction

The scHSCs were generated as previously described [37] based on published protocols for scHSC 2D differentiation [32, 33]. The differentiation was performed on four hPSC lines: WAe001-A (H1) (scHSC_1, WiCell Research Institute), UCSFi001-A (WTC11) (scHSC_2, Corelli Institute for Medical Research), WTSIi046-A (HPSI0214i-wibj_2) (scHSC_3, Wellcome Trust Sanger Institute), and HMGUi001-A (XM001) (scHSC_4, Helmholtz Zentrum München). As we have previously shown that different hPSC lines lead to variations in hPSC-derived HSC models [37], four hPSC lines were used to account for hPSC line-dependent variability. A schematic of the 12-day differentiation protocol is presented in Fig. 1A. See also Additional File 2 for media compositions. The differentiation media was changed every 48 h or every 24 h if a new media composition was required.

hPSCs were seeded as single cells and cultured on plates coated with 1% (v/v) Geltrex (Thermo Fisher Scientific) in E8 media (Thermo Fisher Scientific) supplemented with 10 µM Rock inhibitor (STEMCELL technologies). The differentiation protocol was initiated at approximately 25% confluency after 24–48 h of incubation. On day 0, the cells were incubated in DMEM/ F-12 medium with Glutamax™ supplement (Thermo Fisher Scientific) with 1% (v/v) MEM Non-Essential Amino Acids Solution (Thermo Fisher Scientific) and 1% (v/v) B-27™ Supplement (Thermo Fisher Scientific) and growth factors Activin A at 100 ng/mL (Peprotech), CHIR at 3 µM (Tocris), and BMP-4 at 20 ng/mL (Peprotech). From day 1 to day 5, the cells were grown in a basal mesoderm medium consisting of DMEM/ F-12 medium with Glutamax™ supplement containing 1% (v/v) MEM Non-Essential Amino Acids Solution, 1% (v/v) B-27™ Supplement (Thermo Fisher Scientific), 0.025% Insulin-Transferrin-Selenium (ITS-G) (Thermo Fisher Scientific), 2.5 µM Dexamethasone (Merck Sigma-Aldrich) and 100 µM 2-Phospho-L-ascorbic acid trisodium salt (Merck Sigma-Aldrich). This basal mesoderm media was supplemented with 20 ng/mL BMP-4 on day 1, and 20 ng/mL BMP-4, 20 ng/mL FGF-1 (Peprotech), and 20 ng/mL FGF-3 (R&D Systems) on days 2–5. The differentiating cells were passaged on day 5 and 10 µM Rock inhibitor was added to the differentiation medium for 24 h. Accutase (Thermo Fisher Scientific) was used for detachment and the cells were plated as single cells in a ratio of 1:3 on plates coated with 1% (v/v) Geltrex. From day 6 to day 12, the cells were grown in a basal HSC medium consisting of DMEM/ F-12 medium with Glutamax™ supplement containing 1% (v/v) MEM Non-Essential Amino Acids Solution, 1% (v/v) Fetal Bovine Serum (FBS) (Thermo Fisher Scientific), 0.025% ITS-G, 2.5 µM Dexamethasone, and 100 µM 2-Phospho-L-ascorbic acid trisodium salt. This basal HSC media was supplemented with 20 ng/mL FGF-1, 20 ng/mL FGF-3, 100 µM Palmitic acid (PA) (Merck Sigma-Aldrich), and 5 µM Retinol (ROL) (Merck Sigma-Aldrich) on days 6–12.

### Human pHSC culture

Commercially available pHSCs were purchased from iXCells Biotechnologies (two donors) and BeCytes (one donor) and cultured according to the manufacturer’s instructions on plates coated with 1% (v/v) Geltrex. The pHSCs from iXCells Biotechnologies were used for IF imaging and the radioactive substrate oxidation assays. The pHSCs from BeCytes were used for cytokine multiplex analysis, VA ELISA, and lactate secretion. pHSCs from all donors were used in qPCR analysis and HT LD image analysis. A 24-hour treatment was used on pHSCs from BeCytes since this donor material was sensitive to the starvation and starvation markedly reduced growth.

The pHSCs from iXCells Biotechnologies were initially plated in Stellate Cell Growth Medium (iXCells Biotechnologies) and pHSCs from BeCytes were thawed in NPCs Thawing Medium (All Types) (BeCytes) before culture in Stellate Cell Growth Medium (BeCytes) after 24 h. Treatments were initiated 24–48 h after thawing, see “scHSC and pHSC culture, treatments, and activation”. An aliquot of the pHSCs was collected before plating for downstream transcriptomic analysis.

### scHSC and pHSC culture, treatments, and activation

Differentiated scHSCs and human pHSCs were grown in four conditions: “Control”, “Starvation”, “TGF-β”, and “Starvation + TGF-β”. The “Control” medium consisted of DMEM/ F-12 medium with Glutamax™ supplement containing 1% (v/v) MEM Non-Essential Amino Acids Solution, 1% (v/v) FBS (Thermo Fisher Scientific), 0.025% ITS-G, 2.5 µM Dexamethasone, 100 µM 2-Phospho-L-ascorbic acid trisodium salt, 100 µM PA, and 5 µM ROL. In the “Starvation” and “Starvation + TGF-β” media, PA and ROL supplementation was removed and FBS was substituted for 1% (v/v) Bovine Serum Albumin (BSA) (VWR, SEQENS). In the “TGF-β” and “Starvation + TGF-β” media, TGF-β (Peprotech) was added at a concentration of 25 ng/mL, a concentration used in several scHSC activation models [32, 35, 37]. The treatments were chosen to investigate the individual and combinational effects of short-term ROL and PA depletion and TGF-β-induced activation. See also Additional File 2 for media compositions. The length of the treatments was 48 h if not otherwise stated and media was replenished every 48 h. The 48-hour TGF-β-induced activation model was chosen to avoid adverse effects of long-term cultivation.

### mRNA sequencing by Novogene

Cultured cells were detached with Trypsin-EDTA (Merck Sigma-Aldrich) before total RNA isolation by RNeasy Mini kit (Qiagen). The proper concentration and volume of samples were sent to Novogene UK Cambridge Sequencing Center for mRNA sequencing with WBI-Quantification. Novogene performed RNA sample quality control, mRNA library preparation (poly A enrichment), and Illumina sequencing PE150 (6G raw data per sample). Novogene also performed standard bioinformatics analysis including data quality control and data filtering, mapping to reference genome, gene expression quantification and correlation analysis, differential expression analysis, and enrichment analysis including GSEA enrichment analysis.

### RT-qPCR

RNA from scHSCs was isolated by Trizol™ (ThermoFisher Scientific) and RNA from pHSCs was isolated using RNeasy Micro kit (Qiagen). cDNA was generated using a High-Capacity cDNA Reverse Transcription Kit (ThermoFisher Scientific). Gene expression was subsequently determined by RT-qPCR analysis on a ViiA 7 (ThermoFisher Scientific) thermocycler using TaqMan probes and TaqMan Gene Expression Master Mix (ThermoFisher Scientific). *GAPDH* was used as the housekeeping gene.

### Immunofluorescence confocal microscopy

Cells were fixed on glass slides with 4% paraformaldehyde (PFA) (Merck Sigma-Aldrich) for 10 min. The samples were then permeabilized and blocked in a blocking solution made of 0.1% (v/v) Triton-X (Merck Sigma-Aldrich) and 10% (v/v) Fetal Bovine Serum (Thermo Fisher Scientific) diluted in DPBS (Thermo Fisher Scientific). Next, the cells were incubated overnight at 4 ^o^C with primary antibodies properly diluted (see Materials section) in blocking solution before staining with secondary antibodies, and nuclear staining with DAPI (Thermo Fisher Scientific) was performed in the dark for 1 h at room temperature. The stained cells were then mounted on glass slides with mounting media (5% gelatin in 1:1 distilled water and glycerol). Confocal imaging was performed with an LSM700 (Zeiss, Germany) confocal microscope using standard filter sets and laser lines with a 40x oil immersion objective. Stained samples were stored at -20 ^o^C.

### α-SMA signal intensity analysis (Python)

α-SMA intensity of confocal images was quantified by an automated Python3 (03.09.2012) script. The script is on GitHub: https://github.com/ingridwilhelmsen/a-SMA_analysis. Antibody dilutions and confocal microscope settings were equal for all samples to ensure that the input material was quantifiable.

### Flow cytometry

Flow cytometry was performed at the Flow Cytometry Core Facility at Oslo University Hospital, Gaustad, on a BD LSRFortessa™ (BD Biosciences) Cell Sorter and analysis was performed using FlowJo (10.9.0).

The cells were fixed as single cells by incubation in 4% paraformaldehyde (PFA) (Merck Sigma-Aldrich) for 15 min before blocking in a blocking solution made of 10% (v/v) Fetal Bovine Serum (Thermo Fisher Scientific) diluted in DPBS (Thermo Fisher Scientific) for 30 min. For subsequent steps, cells were washed and stained in a FACS buffer containing DPBS and 0.1% BSA. The cells were stained overnight with 1:50 diluted antibody against PDGFR-β (R&D Systems) before secondary antibody staining with 1:250 diluted Alexa Fluor^®^ 488 AffiniPure Donkey Anti-Goat IgG (H + L) (Jackson ImmunoResearch) for 60 min. The cells were washed two times after each staining. PDGFR-β was detected in the flow cytometer by an Alexa Fluor 488-A laser and autofluorescence was assessed using a Pacific Blue 405 laser with a 450/50 band-pass filter, which is adequate but suboptimal for detection of VA which is autofluorescent at 330 nm [41]. A total of 10,000 cells were counted per sample. hPSC samples stained with only secondary antibody were used as negative controls.

### Cytokine multiplex discovery

Conditioned media from scHSCs was collected after 48 h of treatment if not otherwise stated and stored at -80 ^o^C until the assay was performed. Separate wells in 96-well plates were regarded as technical replicates.

The Human Luminex^®^ Discovery Assay (R&D Systems/Bio-Techne) was used for the detection of 12 selected cytokines (CCL2, CCL3, CCL4, CCL5, CCL21, CXCL1, HGF, IL-1 beta, IL-6, IL-8, IL-10, TNF-alpha) according to the manufacturer’s instructions and the readout was performed with a Bio-Plex 200 (Bio-Rad) reader. Values that were extrapolated outside the range of the assay were excluded from downstream analysis.

### UV autofluorescence image analysis

VA emits autofluorescence at 330 nm and is thus detectable under UV light [41]. Images were acquired using an AxioVert.A1 microscope equipped with a Colibri7 LED light source using the UV line (Excitation: 385/30, Emission: QBP 425/30) of Filter Set 90 HE LED, a 10x A-Plan 0.25 NA objective, and a CMOS digital camera (all from Carl Zeiss). The exposure time was set to 50 ms. To minimize bleaching of the UV signal [41], all images were acquired within 1 s of UV light exposure. Confluent areas were independently chosen under bright field before UV light acquisition. Separate wells in 96-well plates were regarded as technical replicates. The images were analyzed in ImageJ (Java 8, 32-bit) by an automated macro implementing thresholding by using the Triangle method [42] as previously described [37].

### ELISA for Procollagen C-peptide 1

Conditioned media from scHSCs was collected after 48 h of treatment if not otherwise stated and stored at -80 ^o^C until the assay was performed. Separate wells in 96-well plates were regarded as technical replicates.

The Procollagen I C-Peptide ELISA kit (Takara Bio Inc) was used according to the manufacturer’s instructions and the readout was performed by measuring absorbance at 450 nm with a Wallac Victor^2^ 1420 multilabel counter (Perkin Elmer/Wallac) reader.

### ELISA for human vitamin A

Cultured media was removed from the cells after 48 h if not otherwise stated. The cells were then washed with DBPS and subsequently snap-frozen at -80 ^o^C until the assay was performed. Separate wells in 96-well plates were regarded as technical replicates.

The cells were lysed by RIPA lysis and extraction buffer (ThermoFisher Scientific) before intracellular Vitamin A levels were measured by a Human Vitamin A ELISA Kit (Colorimetric) (Novus Biologicals//Bio-Techne) according to the manufacturer’s instructions. The readout was performed by measuring absorbance at 450 nm with a Wallac Victor^2^ 1420 multilabel counter (Perkin Elmer/Wallac) reader.

### Lactate detection

Lactate was measured both on conditioned media and intracellularly on cell lysates from RIPA lysis and extraction buffer lysis. Analysis was performed after 48 h of treatment if not otherwise stated.

A Lactate Glo-Assay (Promega) was used for the detection of lactate according to the manufacturer’s instructions. The readout was performed with a Glomax^®^-Multi + Detection system (Promega).

### Protein quantification

Protein levels were measured to normalize the results obtained from assays measuring cytokine release, Procollagen C-Peptide I release, intracellular Vitamin A, radioactive substrate oxidation, oxygen consumption rate (OCR) and extracellular acidification rate (ECAR), and lactate levels. The results were normalized to 100 mg/mL protein as measured by Pierce™ BCA Protein Assay Kit (Thermo Fisher Scientific) on cell lysates lysed by RIPA lysis and extraction buffer (Thermo Fisher Scientific). For radioactive substrate oxidation, all results were adjusted for protein content, measured by the Bio-Rad protein assay using a VICTOR™ Nivo Multilabel Plate Reader (PerkinElmer).

### Radioactive substrate oxidation assay

The evaluation of glucose and oleic acid metabolism was conducted by radioactive substrate oxidation assay as previously described [43]. Following cell activation, media was replaced by D-[^14^C(U)]glucose (0.5 µCi/ml, 200 µM) or [1-^14^ C]oleic acid (OA) (0.5 µCi/ml, 100 µM) substrates during 4 h and CO_2_ trapping was performed. In brief, a 96-well UniFilter^®^ microplate, activated for the capture of CO_2_ by the addition of 1 M NaOH, was mounted on top of the 96-well cell-cultured plate. After 4 h of trapping, the cells were washed with PBS and harvested in 0.1 M NaOH. The ^14^CO_2_ trapped in the filter and ^14^C remaining in cells (cell-associated (CA)) was measured by the addition of scintillation fluid (Ultima Gold XR) and counted on a 2450 MicroBeta2 scintillation counter. Cellular uptake was counted as the sum of ^14^CO_2_ and CA radioactivity measurements: CO_2_ + CA. Fractional oxidation was calculated as CO_2_/uptake.

### Oxygen consumption rate (OCR), extracellular acidification rate (ECAR), and glycolysis inhibition

The OCR and ECAR were analyzed with a Seahorse XFe24 Analyzer (Agilent). Differentiating scHSCs were plated in XFe 24-well plates (Agilent) on day 5 of differentiation during the mid-differentiation passage.

The cells were washed and incubated at 37 ^o^C in an incubator without CO_2_ with Seahorse XF DMEM assay medium (Matriks AS) supplemented with glucose (10 mM), glutamine (2 mM), and pyruvate (1 mM) for 1 h before the assay. The assay was performed with serial injections of oligomycin (1.5 µM, Cell Signaling Technology), FCCP (1 µM, Merck Sigma-Aldrich), and a mixture of rotenone (0.5 µM, Merck Sigma-Aldrich) and antimycin A (0.5 µM, Merck Sigma-Aldrich). The baseline was determined in the Seahorse assay media during 5 cycles of measurement with 8-minute intervals (3 min mix, 2 min wait and 3 min measurement). The OCR and ECAR values were normalized to BCA values. Unless otherwise stated, the OCR and ECAR values of each scHSC lineage were normalized to the baseline of the “Control” condition of each respective cell line – resulting in the unit “% of baseline” – to allow for comparison of the results.

2-Deoxy-D-glucose (2-DG, Merck Sigma-Aldrich) was used at a final concentration of 50 mM to inhibit glycolysis. 2-DG was injected using the Seahorse XFe24 Analyzer during 5 cycles of measurement with 8-minute intervals (3 min mix, 2 min wait and 3 min measurement) before serial injection of oligomycin, FCCP, and Rotenon/ Antimycin A to assess the effect of 2-DG on the basal mitochondrial respiration.

### Holotomographic lipid analysis

**Fig. 2:**
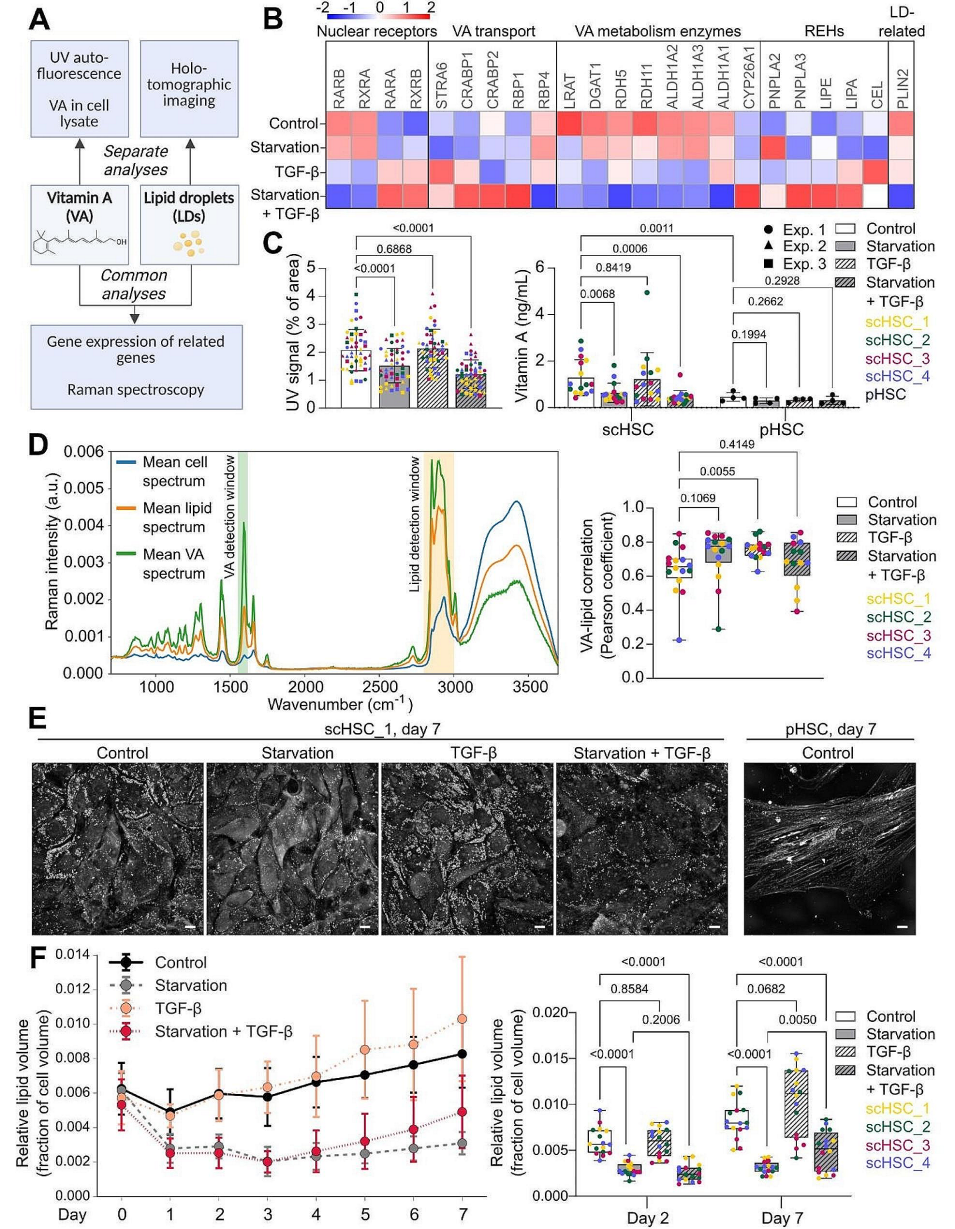
Vitamin A (VA) and lipid droplets (LDs) during human scHSC starvation and activation. (**A**) Schematic representation of the methods used for the analysis of VA and LDs. VA representative molecule: Retinol. (**B**) Heatmap showing the z-score of selected VA- and LD-related genes from mRNA sequencing data of scHSCs. *n* = 4 cell lines, with *N* = 3 technical replicates. (**C**) VA storage was indirectly measured by quantifying the UV signal of fluorescence images of scHSCs (48 h) and directly measured by ELISA of cell lysates of scHSCs (48 h) and pHSCs (24 h). UV imaging: Three independent experiments with *n* ≥ 3 cell lines per experiment, *N* = 4 technical replicates, ELISA: scHSCs: *n* = 4 cell lines, *N* = 3 technical replicates, pHSCs: *n* = 1 donor, *N* = 4 technical replicates. (**D**) Raman spectra of a “control” sample of scHSC_1 and subsequent VA-lipid correlation analysis of scHSCs after 48 h of treatment. *n* = 4 cell lines, *N* = 4 randomly selected ROI scanned. (**E**) Representative holotomographic (HT) images of scHSC_1 and pHSCs after 7 days of treatment. Scale bars: 10 μm. (**F**) Lipid volume per cell volume of scHSCs during 7 days of treatment as quantified in HT images. Data from days 2 and 7 are also shown as box plots. *n* = 4 cell lines, *N* = 4 randomly selected regions of interest (ROI) imaged. Statistical tests: Unpaired t-test with Welch’s correction

Holotomographic (HT), live-cell 3D imaging for the detection of lipids was performed using the Tomocube HT-X1 system that measures refractive index as imaging contrast. HT imaging allows live identification of subcellular 3D structures based on their refractive index with unprecedented resolution [44]. Lipids have a high refractive index and are therefore easily identified and quantified in HT imaging (representative images in Fig. 2E). The live cells were imaged in Cellvis 6 or 12 Well Glass Bottom Plates (Cellvis) and images were segmented using a Machine Learning (ML) model (XGBoost). The model was trained on a sub-set of the images where cells were segmented from the background using the local sharpness of the image as computed by a local 2D laplacian operator. The ML model was trained and validated on 60% and 20% of the already segmented data respectively and the threshold for positive detection was set as per maximizing the F-score. Testing was performed on the remaining 20% of the images and led to an area under the receiver operating characteristic (AUROC) of 0.997, ensuring good detection.

Lipid droplets, which present a relatively higher refractive index compared to the rest of the image, were detected using a 3D Laplacian of Gaussian on the segmented images to detect the spherical volumes of higher refractive index which are typical of lipid droplets.

### Mitochondria analysis

The mitochondria were stained for 30 min using MitoTracker™ Deep Red FM - Special Packaging (ThermoFisher Scientific) at a dilution of 1:5000 (200 nM) according to the manufacturer’s instructions. The fluorescent signal was then imaged with the Tomocube HT-X1 system.

Mitochondria were first segmented using a standard threshold whose value was computed by Otsu’s algorithm [45] on each stack of images and then skeletonized using Zhang’s algorithm [46]. Skeletonized stacks were labeled (connectivity of three) and the mean size and number of resulting clusters were quantified.

### Confocal Raman spectroscopy

Cells grown on glass coverslips were treated as indicated for 48 h, fixed (4% PFA, 10 min), and kept in PBS. Raman spectra were recorded using a confocal Raman microscope (alpha300R, WITec) equipped with a 75 mW 532 nm laser (approximately 58.4–59.2 mW at the sample), a spectrometer (UHTS300S, WITec) with a 600 groove/mm grating and a thermoelectrically cooled back-illuminated charge-coupled device camera (Oxford Instruments - Andor) using a ×63/1.0 NA water-immersion objective lens (W Plan-Apochromat, Zeiss). Cells were scanned using a Large Area Scan of 200 × 200 µM with 200 points and 200 lines using an integration time of 0.5 s and Topography correction.

Pre-processing of the scans was performed in Python3 by first removing the cosmic rays using a local median filter and the baseline using an asymmetrically reweighted penalized least squares smoothing (arPLS) [47] as implemented by pybaselines [48] which was computed on the mean spectrum of each scan. Every spectrum was renormalized so that the integral of the signal over the full wavenumber range was equal to unity.

Amounts of VA and lipids were estimated by the integrals of the Raman signal in the spectral windows of [1554, 1615] cm^− 1^ [49, 50] and [2800, 3000] cm^− 1^ [51, 52] respectively (see Fig. 2D, left). The correlation between these two compounds was estimated using Pearson’s correlation coefficient across the whole scans.

### Statistical analysis

Independent cell lines are treated as biological replicates, denoted “n”, and separate wells within the same differentiation and cell line are treated as technical replicates, denoted “N”.

Statistical significance was determined using the statistical analysis unpaired t-test with Welch’s correction in the GraphPad Prism (10.2.0) software. When stated, outliers were identified and removed from analysis using ROUT, Q = 1%.

Results presented as bar graphs are expressed as mean ± standard deviation (SD). When values are mentioned in-text, they are stated as mean ± SD. Boxplots visually represent the five-number summary of the data, encompassing the minimum and maximum values with the whiskers, the first and third quartiles (Q1 and Q3) of the data within the box, and the median (Q2) at the line in the box. The error bars on line graphs represent the SD unless otherwise stated. All data points used to make the bar graphs and boxplots are visualized in the figures. Data from the different cell lines are colored as follows throughout the figure: Yellow for scHSC_1, green for scHSC_2, magenta for scHSC_3, and blue for scHSC_4.

All *p*-values are written on the graphs. In general, *p*-values ≤ 0.05 were considered of interest. In enrichment analysis of mRNA seq data, FDR q-values ≤ 0.25 were considered of interest.

### Figure creation

The schematic figures were created with BioRender (biorender.com). Graphs were created with GraphPad Prism (10.2.0) or using Python3 (03.09.2012).

## Results

### Starvation of ROL and PA combined with TGF-β enhances the activation-related transcriptomic profile of scHSCs

scHSCs were differentiated from four hPSC lines as previously described [37] based on published approaches for scHSC 2D differentiation [32, 33]. To ensure the quality of the differentiated scHSCs, gene expression analysis by RT-qPCR showed downregulation of the pluripotency marker octamer-binding transcription factor 4 (*OCT4*), and expression patterns similar to previous scHSC reports [32, 37] of the HSC markers platelet-derived growth factor β (*PDGFR- β*), neural adhesion molecule (*NCAM1*), *desmin*, activated leukocyte cell adhesion molecule (*ALCAM*), lecithin retinol acyltransferase (*LRAT*), and actin alpha 2 (*ACTA2*) (Figure S1A [Additional File 1]). Protein expression of the HSC-typical markers PDGFR- β, NCAM1, and vimentin was confirmed by IF using confocal imaging (Figure S1B [Additional File 1]), while lipid accumulation was observed throughout the differentiation by HT imaging (Figure S1C [Additional File 1]).

Following the scHSC differentiation, scHSCs (i) continued to receive ROL and PA, hereafter referred to as “control”, (ii) were starved for ROL and PA, hereafter referred to as “starved” or “starvation”, in (iii) the presence or (iv) absence of the potent HSC activator TGF-β for 48 h unless otherwise stated (Fig. 1A). To ensure that the four treatment conditions did not alter the HSC identity of the cells, all scHSC populations were confirmed to express high levels (> 91%) of the HSC surface marker PDGFR- β [53, 54] as shown by flow cytometry (Figure S1D [Additional File 1]).

To evaluate the effect of the aforementioned treatments on the transcriptome of the scHSCs, mRNA sequencing (Novogene) on cells from all four hPSC-derived scHSC lineages was performed. The principal component analysis (PCA) showed that all four scHSCs lineages under control and starvation conditions had similar transcriptomic patterns, suggesting minimal changes solely due to ROL and PA starvation in the 48-hour timeframe of the assay (Fig. 1B; see also Figures S2A and S2B). However, treatment with TGF-β consistently resulted in distinct clustering along the PC2 axis indicating that the TGF-β treatment led to significant alterations in the dataset. Notably, the combination of starvation and TGF-β led to a further separation of the clustering for all cell lines, most evident in scHSC_1 and scHSC_2. This observation suggests that the depletion of ROL and PA alone has a limited impact on the HSC transcriptomic profile, however, the depletion has a synergistic effect in combination with the activation-inducer TGF-β.

To further delve into this observation, we examined the expression of specific genes associated with TGF-β signaling, HSC activation, and cytokine secretion (Fig. 1C). The analysis revealed activation of TGF-β signaling and typical HSC activation following TGF-β treatment, which was amplified when combined with starvation. This effect was confirmed on selected genes by RT-qPCR (Figure S2C [Additional File 1]) and was also reflected in Wnt [55], Hippo [56], and TGF-β/ Smad2/3 signaling connected to HSC activation (Figures S2D and S2E [Additional File 1]), further demonstrating the synergistic effect of ROL and PA starvation combined with TGF-β. Interestingly, the combination of starvation and TGF-β treatment also resulted in alterations in the expression profile of cytokines compared to TGF-β treatment alone.

Subsequently, we investigated whether the observed changes in gene expression in the treatment groups were translated to the corresponding proteins implied in HSC activation. As anticipated, image analysis of IF-stained α-smooth muscle actin (α-SMA, encoded by the *ACTA2* gene), a common HSC activation marker associated with hepatic fibrosis [57], revealed an increase of α-SMA stress fibers upon treatment with TGF-β alone and in combination with starvation. (Figure 1D and E). Procollagen type I C-peptide, a classical HSC activation marker [5] encoded by the *COL1α1* gene, displayed a modest increase upon treatment with TGF-β as measured by ELISA, however, not under starved conditions (Fig. 1F). However, surprisingly after 48 h of treatment, the release of cytokines in the cell culture media as measured by cytokine multiplex assays did not correspond to the altered gene expression levels under the same conditions (Fig. 1F). In contrast, cytokine release was generally unaltered or lowered in the treatment groups. This observation was largely recapitulated in pHSCs (Figure S2F [Additional File 1]), further validating the findings in scHSCs.

In summary, the combined treatment of starvation and TGF-β demonstrated a synergistic effect on the expression of genes associated with HSC activation, implying that the absence of ROL and PA enhances the activation status of scHSCs on the transcriptional level. Additionally, this treatment led to alterations in the transcriptomic profile of cytokines, possibly due to the involvement of VA in inflammatory responses. However, at the given time point for the measurements (48 h), the transcriptomic changes were not translated to increased cytokine secretion either in scHSCs or pHSCs. This could indicate that 48 h post-treatment is a suboptimal time point for cytokine measurements.

### Vitamin A and lipid droplet loss is observed in response to ROL and PA starvation and is not affected by isolated TGF-β-induced activation

The storage of VA in cytoplasmic LDs is considered a defining characteristic of quiescent HSCs in the healthy liver and, correspondingly, the loss of VA and LDs is considered a hallmark of HSC activation [1, 6, 54]. To explore the relationship between VA and LD loss and HSC activation, we investigated the impact of ROL and PA starvation and the activation by TGF-β on VA and LD storage by several methods (Fig. 2A).

First, the effect of the treatments was assessed on selected VA- and LD-related genes (Fig. 2B; see also Figure S3A [Additional File 1]). The nuclear retinoic acid receptors *RARβ* and *RXRα* were downregulated in the TGF-β groups in correspondence with previous studies on human liver disease patients [58, 59]. We also detected a TGF-β-induced increase in genes related to VA transport and a general decrease in genes related to VA metabolism, suggesting that TGF-β exposure at the measured time point leads to a switch from VA storage to mobilization and utilization. Secondly, as liver disease is associated with impaired VA homeostasis and retinol mobilization from LDs through increased retinyl ester hydrolase (REH) activity [60], we investigated the gene expression of enzymes with REH activity that have been suggested as HSC-relevant. Several REH enzymes (patatin-like phospholipase domain containing 2 (*PNPLA2)* [61], *PNPLA3* [38], lipase E hormone-sensitive type (*LIPE/HSL)* [62], lipase A lysosomal acid type *(LIPA/LAL)* [13, 63], and carboxyl ester lipase (*CEL)* [64]) were upregulated during TGF-β-induced activation, particularly when combined with starvation, suggesting an increase in mobilization of VA from LD. Lastly, consistent with the literature on human pHSCs [65], the gene encoding the LD-coating and lipolysis-related protein Perilipin 2 (*PLIN2/ADRP*) was downregulated during starvation combined with TGF-β, suggesting an increased breakdown of LDs.

Next, the VA content was measured at the specified time points (i) indirectly by quantifying UV positivity and (ii) directly by quantifying total VA in cell lysates by ELISA (Fig. 2C). Quantification of UV positivity both by image analysis (Fig. 2C, left) and by flow cytometry (Figure S3C [Additional File 1]) revealed that while starvation led to a reduction in the UV signal, surprisingly, activation by TGF-β alone did not. The same pattern was observed when measuring total VA content (Fig. 2C, right).

Interestingly, pHSCs did not produce UV signals detectable by imaging (Figure S3B [Additional File 1]) and contained significantly less intracellular VA as measured by ELISA compared to the scHSCs (Fig. 2C, right), containing approximately 36% VA of scHSCs when comparing averages (1.290 ± 0.771 µg/mL for scHSCs, 0.4602 ± 0.185 µg/mL for pHSCs, the values are reported as mean ± standard deviation). Additionally, the VA level in the pHSCs was unaffected by the TGF-β and starvation treatments. Together, this suggests that pHSCs may be suboptimal for studying VA alterations during HSC activation while scHSCs can be grown for relatively longer periods and show VA levels that are sensitive to interventions.

We next employed confocal Raman spectroscopy to investigate the correlation between the VA and lipid signals estimated as the integral in their respective wavenumber ranges (Fig. 2D, left). The analysis revealed a significant spatial correlation between the broad VA signal and lipid signal in all treatment groups, indicating their co-localization and supporting the application of LDs as an indirect measure of VA content in HSC (Fig. 2D, right; see also Figures S4A and S4B). We also observed a small increase in the VA-lipid correlation after treatment with TGF-β, possibly indicating a spatial change in VA storage in response to the activation stimulus.

Since the Raman analysis demonstrated a clear correlation between VA and lipids, we lastly analyzed changes in lipid content in the scHSCs during prolonged treatment over 7 days as an indirect method to both validate earlier observations (48-hour time point) and to track further developments of LDs. For this, all four scHSC lines were monitored using live cell HT imaging every 24 h (representative images in Fig. 2E). The relative lipid volume of the cells was expectedly reduced by starvation, however, it was unaffected by TGF-β during the first four days of treatment (Fig. 2F, left). However, by day 7, treatment with TGF-β had increased the relative lipid content both in the presence and absence of starvation (Fig. 2F, right). Notably, human pHSCs contained lower lipid contents than the scHSCs and were, similar to our earlier observations of VA, unresponsive to the treatments (Figure S4C [Additional File 1]).

In summary, both VA and LD loss in our experimental system was observed only in response to ROL and PA starvation and both VA and LDs were unaffected by TGF-β treatment alone. Additionally, we have demonstrated that commercially available pHSCs are not ideal for studying VA and LD alterations during HSC activation in vitro while scHSCs have VA levels and LDs that are sensitive to interventions.

### The energy metabolism of scHSCs is altered by TGF-β-induced activation, which reduces glycolysis, and starvation, which reduces β-oxidation

**Fig. 3:**
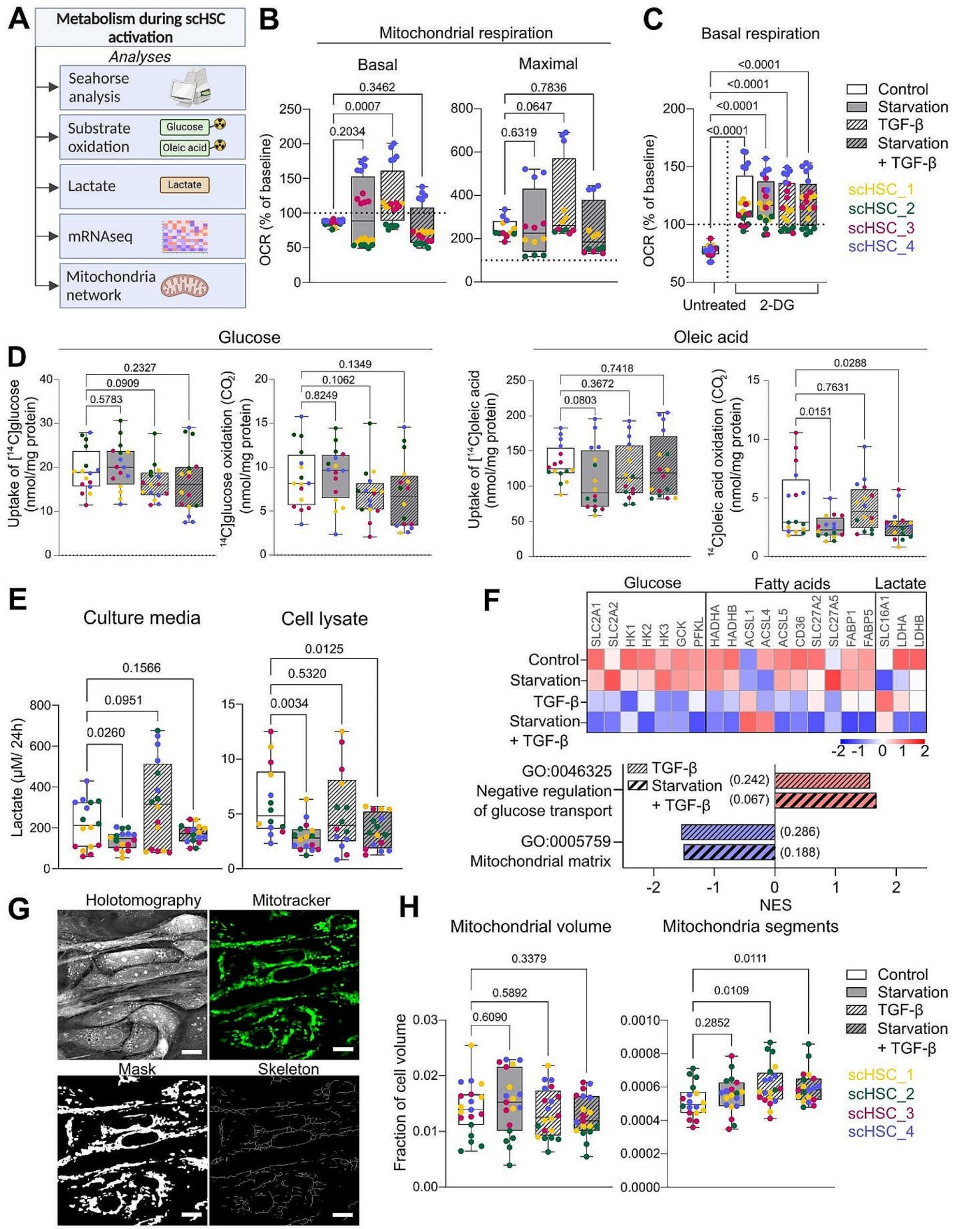
Metabolism during ROL and PA starvation and TGF-β-induced activation in scHSCs. (**A**) Schematic representation of the methods used for the analysis of the metabolism of scHSCs. (**B**) Basal and maximal mitochondrial respiration of scHSCs as measured by oxygen consumption rate (OCR). The horizontal dotted lines indicate the baseline set to 100%. *n* = 4 cell lines, *N* ≥ 2 technical replicates. (**C**) Basal mitochondrial respiration of scHSCs in the presence and absence of 2-DG as measured by OCR. The 2-DG values were normalized to the pre-treated scHSCs of the given cell line and condition to evaluate 2-DG-induced changes. The horizontal dotted line indicates the baseline set to 100%. *n* = 4 cell lines, *N* ≥ 2 technical replicates. (**D**) Glucose and oleic acid uptake and oxidation in scHSCs after 48 h of treatment. *n* = 4 cell lines, *N* = 4 technical replicates. (**E**) Lactate in culture media and cell lysates of scHSCs per 24 h, after 48 h of treatment. *n* = 4 cell lines, *N* = 4 technical replicates, cleaned for outliers using ROUT, Q = 1%. (**F**) Heatmap showing the z-score of selected metabolism-related genes and Gene Ontology (GO) terms upregulated (red) and downregulated (blue) compared to “Control”. Numbers in parentheses show the false discovery rate (FDR) q-value. NES: Normalized enrichment score. *n* = 4 cell lines, with *N* = 3 technical replicates. (**G**) Representative holotomographic- and fluorescence images (MitoTracker-stained) of scHSC_1, as well as the computed mitochondrial mask and skeleton. Scale bars: 10 μm. (**H**) The mitochondrial volume and the number of mitochondrial segments computed from 3D fluorescence live-cell imaging (mitotracker-stained), normalized by the cell volume. *n* = 4 cell lines, *N* = 5 randomly selected regions of interest (ROI) imaged. The data was cleaned for outliers using ROUT, Q = 1%. Statistical tests: Unpaired t-test with Welch’s correction

HSC activation is a metabolism-altering process. Therefore, we analyzed the metabolism of the scHSCs by measuring oxygen consumption rate (OCR) and extracellular acidification rate (ECAR), glucose and oleic acid uptake and oxidation, lactate production, relevant transcriptomic data, and mitochondrial network analysis in response to activation by TGF-β and starvation of ROL and PA (Fig. 3A).

OCR measurement was performed to assess the mitochondrial activity of the scHSCs after 48 h of treatment. Treatment with TGF-β alone led to an increase in the basal mitochondrial respiration and to a trend of enhanced maximal mitochondrial respiration (Fig. 3B; see also Figure S5A [Additional File 1); however, there was significant variation between the scHSC lineages. Additionally, the glycolysis inhibition by 2-Deoxy-D-Glucose (2-DG) was employed to elucidate the ability of the scHSCs for mitochondrial compensation upon glycolysis blockage. The glycolysis inhibition was confirmed by reduced ECAR upon 2-DG injection (Figure S5A [Additional File 1]). Interestingly, we detected an increase in the basal mitochondrial respiration in all treatment groups in response to 2-DG, suggesting a metabolic flexibility in the scHSC material (Fig. 3C).

Glucose and fatty acid metabolism were then investigated by radioactive substrate oxidation assays, which employ a more direct measure of the metabolic pathways than the OCR analysis. Treatment with TGF-β led to a small decrease in glucose uptake reflected in a reduction of glucose oxidation (Fig. 3D; see also Figure S6A [Additional File 1]), observations which were statistically significant upon experimental repeats (Figure S6B [Additional File 1]). The oleic acid uptake and oxidation were unaffected by the treatment with TGF-β. However, oleic acid oxidation was reduced in the starved conditions irrespective of treatment with TGF-β.

To further examine glucose metabolism, we investigated the lactate levels in culture media and cell lysates to assess anaerobic glycolysis (Fig. 3E). Previously, activation of cultured mouse HSCs has been reported to increase intracellular lactate levels [19]. However, we were only able to detect a trend of increased lactate levels in the culture media during treatment with TGF-β. The same trend was mirrored in ECAR data – indirectly measuring lactate by increased acidification – where TGF-β treatment led to a trend of increased ECAR (Figure S5A [Additional File 1). Notably, starvation reduced both secreted and intracellular lactate independently of TGF-β, a result that was reproduced in human pHSCs (Figure S7A [Additional File 1]). Hence, starvation reduces the anaerobic glycolysis in our model.

Next, we examined the gene expression of genes related to the transportation and metabolism of glucose, fatty acids, and lactate (Fig. 3F). We observed that treatment with TGF-β generally downregulated glucose- and fatty acid-related genes. Notably, this was the case for glucose transporters 1 and 2 (*GLUT1/ SLCA2A1* and *GLUT2/ SLCA2A2*), which are transmembrane proteins responsible for glucose transportation into the cell [66]. Correspondingly, Gene Ontology (GO) enrichment analysis revealed that negative regulation of glucose transport was indeed enriched in both TGF-β-treated groups compared to the control. Downregulated expression of *GLUTs* and glucose transport could explain the lowered uptake of glucose during treatment with TGF-β. Similarly, starvation generally resulted in the downregulation of the lactate transporter monocarboxylate transporter 1 (*MCT1/ SCLA16A1*) as well as lactate conversion enzymes lactate dehydrogenase A and B (*LDHA* and *LDHB*), which could contribute to the effect of starvation on lactate production and release. Lastly, GO analysis revealed a reduced enrichment of mitochondrial matrix in both TGF-β-treated groups compared to control, which prompted us to further investigate the mitochondrial network.

The mitochondria were fluorescently labeled and imaged in 3D using the fluorescent module of the Tomocube HT-X1 (Tomocube), and the resulting images were used to characterize the mitochondrial network (Fig. 3G). The analysis revealed that while the mitochondrial volume was unchanged between the groups, the number of mitochondrial fragments significantly increased upon treatment with TGF-β (Fig. 3H; see also Figure S7B [Additional File 1]). This suggests that TGF-β-induced activation increased the rate of mitochondrial fission, which is the process of mitochondrial division [67].

In summary, the results suggest that in our model, glycolysis is downregulated during TGF-β-induced activation, while starvation leads to reduced oleic acid oxidation and lactate production. The mitochondrial network was more segmented during activation, indicating increased rates of mitochondrial fission.

### Starvation of ROL and PA reduces endoplasmic reticulum stress in scHSCs

**Fig. 4:**
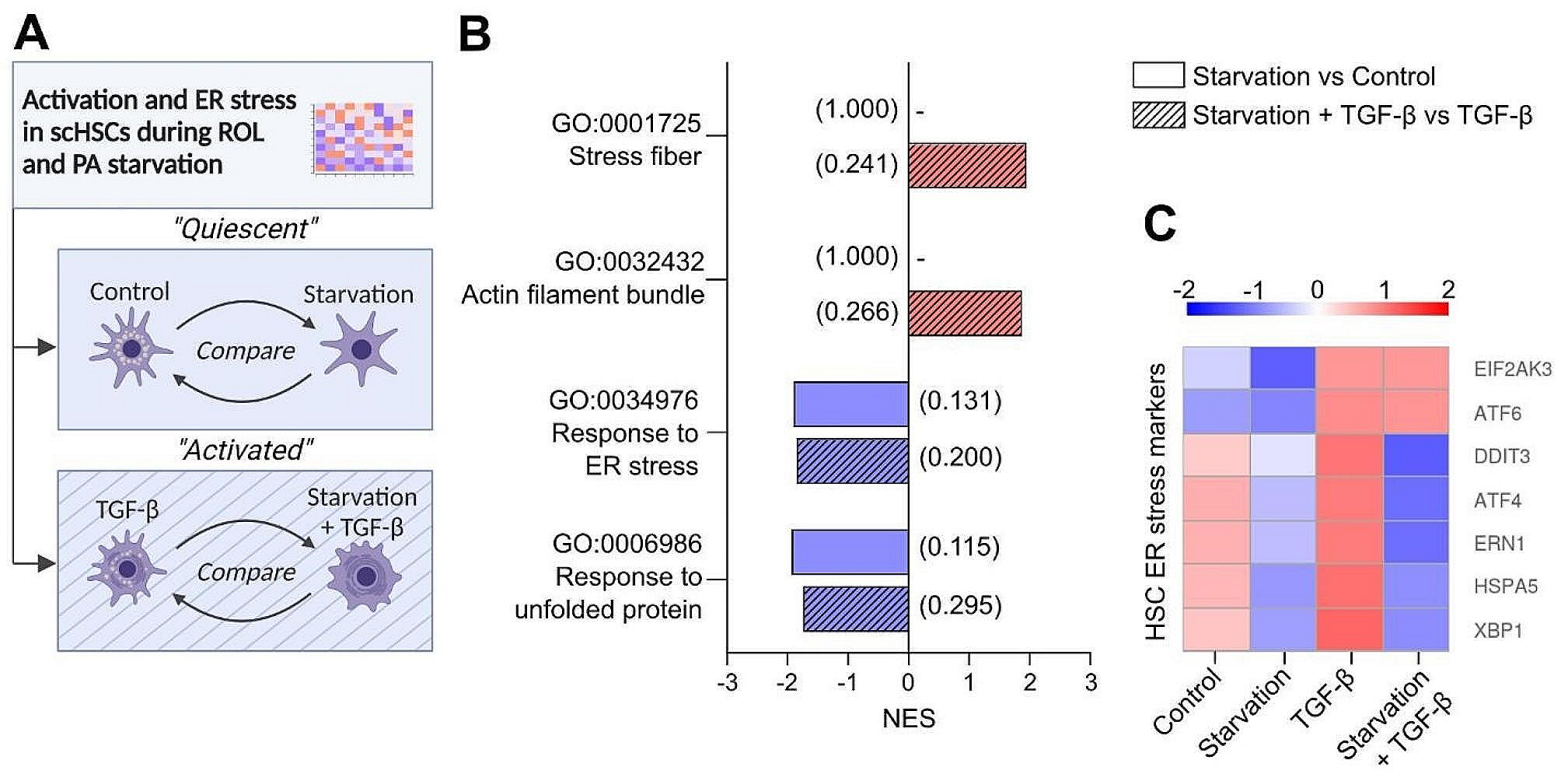
Endoplasmic reticulum (ER) stress during the activation of human scHSCs. (**A**) Schematic outline of the comparisons made in the mRNA sequencing data. (**B**) Gene Ontology (GO) terms upregulated (red) and downregulated (blue). Numbers in parentheses show the FDR q-value. NES: Normalized enrichment score. GO: Gene ontology. *n* = 4 cell lines were sequenced, with *N* = 3 technical replicates per condition. (**C**) Heatmap showing the z-score of selected HSC-typical ER stress-related genes from mRNA sequencing data. *n* = 4 cell lines were sequenced, with *N* = 3 technical replicates per condition

Mouse studies reveal that prolonged in vitro HSC activation can induce endoplasmic reticulum (ER) stress, leading to initiation of the unfolded protein response (UPR) to meet the increased protein demand [68–70]. Similarly, ER stress markers have been reported as elevated in human fibrotic livers and human pHSCs [70, 71]. Consequently, both ER stress and the UPR are considered early indicators of HSC activation. Since our data suggests that starvation with ROL and PA reduces protein (illustrated in procollagen I release (Fig. 1F) and lactate production (Fig. 3E)) while simultaneously increasing activation-related markers when combined with TGF-β (Fig. 1C), we investigated the effect of starvation on the transcriptome of scHSCs in the presence or absence of TGF-β (Fig. 4A).

Enrichment analysis revealed that combining starvation with TGF-β led to the enrichment of activation-related gene ontology (GO) terms “Stress fiber” and “Actin filament bundle” compared to TGF-β alone (Fig. 4B). However, in the absence of TGF-β, starvation did not enrich these terms, further suggesting that starvation of ROL and PA alone does not lead to activation-related transcriptomic changes. Interestingly, we detected downregulation of GO terms related to both ER stress and unfolded proteins in response to starvation independently of TGF-β. These results were reflected in reported HSC ER stress markers, as starvation led to a relative reduction of DNA damage-inducible transcript 3 (*DDIT3/ CHOP)* [68, 70], activating transcription factor 4 (*ATF4*) [68, 69], endoplasmic reticulum to nucleus signaling 1 (*ERN1/ IRE1*) [69], heat-shock protein family A (Hsp70) member 5 (*HSPA5/ BIP*) [68, 69], and X-box binding protein 1 (*XBP1*) [68, 69, 71] (Fig. 4C).

While further investigations are needed, these results suggest that ROL and PA starvation leads to a decreased response to ER stress and UPR both in the presence and absence of activation-inducing stimulus by TGF-β.

## Discussion

HSCs are key players in the development of liver fibrosis, highlighting the importance of understanding the biology of quiescent HSCs in the healthy liver and activated HSCs in fibrotic tissue.

Although the loss of VA-containing LDs is a known hallmark of HSC activation [1, 6], little is known about the exact mechanisms and interplays of the processes. Our work is the first study to extensively assess the impact of VA and PA and TGF-β-driven activation on human scHSCs, using a plethora of methods to establish a solid foundation for future human in vitro HSC studies.

We demonstrate the suitability of human scHSCs for the in vitro fibrosis modeling toolbox. In this study, out of the four applied cell lines, we do not identify a single cell line to consistently act as an outlier across experimental methods, indicating that the use of several cell lines in stem cell-derived models is advantageous for reducing cell line-dependent variability. We demonstrate that treatment with TGF-β leads to expected changes in the transcriptome of the scHSC (Fig. 1C), as well as the formation of α-SMA stress fibers and secretion of procollagen type I (Fig. 1D and E, and 1F). Moreover, the scHSCs were similar to commercially available human pHSCs, as shown in comparable results on gene expression (Figure S1A [Additional File 1]), IF protein detection (Figure S1B [Additional File 1]), and cell energy metabolism (Fig. 3D and S6B). We show that when examining VA and LD content and composition, scHSCs prove to be a superior model in comparison to pHSCs based on VA UV image analysis (Fig. 2C, left, and S3B), VA ELISA (Fig. 2C, right), and HT LD analysis (Fig. 2E and S4C).

Our work adds new dimensions to the accepted dogma that loss of VA and LDs is a defining characteristic of HSC activation. We have previously reported that TGF-β could reduce the autofluorescent VA signal in scHSCs [37]. However, a more extensive analysis of VA content using the combination of autofluorescence detection (in UV image analysis and flow cytometry) with ELISA of intracellular VA content demonstrated that treatment with TGF-β in the presence of ROL and PA media supplementation does not cause VA loss (Fig. 2C). Furthermore, HT LD analysis showed that TGF-β treatment increased the total LD fraction of the cells after prolonged treatment of seven days (Fig. 2F). These results are in contrast with the widespread consensus that connects VA and LD loss to HSC activation both in vivo [7, 8, 12] and in vitro [12, 13]. However, it is noteworthy that most in vivo studies are analyzing longer term pro-fibrotic exposure, where they demonstrate reduced VA levels in hepatic tissue and not specifically in HSCs [7, 8, 14], while increased levels of retinoic acid have been demonstrated in vivo on mouse liver [7, 8] and in vitro on cultured mouse HSCs [72] during early activation. Additionally, a study of both in vivo- and in vitro-activated rat HSCs demonstrated retention of LDs by IF lipid staining and electron microscopy, suggesting that classical lipid staining methods may result in false-negative LD detection. It is also notable that in vitro HSC studies commonly achieve activation through prolonged cultivation, which can have adverse effects on the culture [37] and yield dissimilar results to TGF-β-induced activation. Our work supports both in vivo and in vitro data that indicates a retention of VA and LD in the early phases of HSC activation, highlighting the need for validation of the existing literature with new techniques like label-free HT imaging and analysis - eliminating potential biases that could influence classical staining-based methods - in a variation of activation modalities.

Furthermore, our study reveals surprising results on cell energy-related metabolism during TGF-β-induced activation. Firstly, we demonstrate that the scHSCs have a mitochondrial flexibility, as they are capable of compensation by increasing their basal mitochondrial respiration upon glycolysis inhibition by 2-DG (Fig. 3C). Secondly, in accordance with an in vitro study on mouse HSCs [17], we detect a higher number of mitochondria segments during TGF-β-induced HSC activation (Fig. 3H), suggesting that activation enhances mitochondrial fission. In the same study, the authors linked the enhanced mitochondrial fission to increased OxPhos during HSC activation as observed by increased basal and maximal mitochondrial respiration during treatment with TGF-β. These observations are reflected in our data (Fig. 3B). Importantly, in both this and the aforementioned study, the HSCs were activated by TGF-β treatment, while a contradicting study on rat HSCs reported an increased mitochondrial fusion in HSCs upon activation by prolonged in vitro cultivation [18]. Moreover, we show that TGF-β treatment leads to reduced uptake and subsequent oxidation of glucose (Fig. 3D), in contrast to a study on prolonged culture-activated rodent HSCs and LX-2 cells, which demonstrated increased glucose uptake and OCR as measured by Seahorse analysis [20]. In our study, the functional data obtained by radioactive substrate oxidation assays corresponded to the transcriptional reduction in glycolysis-related genes and glucose- and lactate transporters (*GLUTs/ SLCAs*) in activation (Fig. 3F). Here, too, reports from prolonged culture-activated rodent HSCs have demonstrated the opposite (mRNA detected by RT-qPCR, proteins by IF and Western blotting) and suggested that the shift to aerobic glycolysis is a hallmark of HSC activation [19]. It is of note that our study and the aforementioned studies diverge in model species, activation modality, and detection method, all of which are significant variables. It is possible that the TGF-β activation modality directly impacts the mitochondria, as a recent study on LX-2 cultures shows TGF-β-induced mitochondrial dysfunction by increased levels of ROS and decreased ATP content [73], which could explain the metabolic differences between culture-activated and TGF-β-activated HSCs. Our detection of TGF-β-induced increase in mitochondrial fission supports this. However, ATP production should be tested to validate this hypothesis. Hence, we illustrate the importance of exercising caution when generalizing research results that have not yet been reproduced using several methods or verified in human models, especially since studies on HSC glucose regulation to date are almost exclusively performed in vitro [3].

We show that ROL and PA starvation of scHSCs leads to a phenotype of reduced VA and LD stores (Fig. 2C and E), however, the starvation did not lead to HSC activation, corresponding with studies on LD-deficient mice [74]. At the same time, when combined with pro-fibrotic stimulus by TGF-β, ROL and PA starvation enhanced the activation-related transcriptomic profile of the scHSCs (Fig. 1C), further agreeing with studies on rodent HSCs [75, 76] and human pHSCs [38, 39] where VA and/ or lipid supplementation alleviated activation-associated parameters. Regarding this aspect, our study on human scHSC validates data from rodent models. Our results support the conclusion that VA and LD loss is not in itself sufficient for HSC activation, and that transcriptional attenuation of fibrosis-related markers in response to ROL and PA supplementation is reproducible in human scHSCs in vitro cultures.

Lastly, we observe that ROL and PA starvation affected the cell energy metabolism of scHSCs. Starvation reduces the fatty acid oxidation of the culture (Fig. 3D), suggesting a decreased mitochondrial activity, and lactate production (Fig. 3E). This is important as rodent in vitro studies have linked enhanced OxPhos [17, 18] and increased intracellular lactate levels [19] to the culture-activation of HSCs. Collectively, these observations could indicate that the loss of VA and LDs combined with TGF-β, while pro-fibrotic on the transcriptional level, may lead to metabolism-related changes that attenuate HSC activation. However, it is notable that the substrate oxidation assay used for the detection of metabolic changes only monitors the radiolabeled oleic acid substrate that was added to the culture. It is therefore possible that at the time point of the assay (48 h), the starved conditions were preferentially utilizing their own LD stores for fatty acid oxidation as supported by reduced LD stores seen by HT image analysis (Fig. 2F). Subsequently, the radiolabeled oleic acid may have been redirected to storage rather than oxidation, explaining the observed reduction in radiolabeled oleic acid oxidation. Furthermore, we detected a starvation-induced reduction in transcriptional ER stress and UPR markers (Fig. 4B and C). Several studies have demonstrated a connection between HSC activation, ER stress, and the UPR, however, without consensus in conclusions. On one hand, in vitro rodent models have shown that the UPR is a hallmark of early HSC activation and a driver of fibrosis [68–71, 77], while other studies have shown that the UPR can initiate HSC apoptosis and, hence, resolution of fibrotic development [78–80]. While the exact role of ER stress and the UPR needs further examination, we report that ROL and PA starvation may be an additional factor in these processes.

## Conclusions

In summary, we conducted a comprehensive, systematic in vitro study of the effects of ROL and PA starvation and TGF-β-induced activation on human scHSCs using a plethora of methods. Corresponding with previous studies, we report that while starvation alone does not lead to HSC activation, when combined with TGF-β we observe increased activation-related transcriptomic changes. We also demonstrate a link between ER stress and starvation, as starvation reduces ER stress markers that are reported as activation-inducing. Strikingly, we show that VA and LD contents are retained in TGF-β-activated cultures, calling for a reassessment of the reported correlation between VA and LD loss and in vitro HSC activation by updated methodologies. Similarly, activation by TGF-β resulted in reduced glycolysis and mitochondrial fission, contrasting literature on rodent culture-activated HSCs. We hence highlight the importance of evaluating the relevancy of the HSC activation modality of in vitro systems. The results collectively provide an extensive framework to bridge the gap between rodent and human HSC studies and call for further evaluation of in vitro HSC activation modalities as a step toward testing potential therapeutic interventions.

### Electronic supplementary material

Below is the link to the electronic supplementary material.

Supplementary Material 1

Supplementary Material 2

Supplementary Material 3

## Data Availability

The data supporting the results and analyses in this article will be shared upon reasonable request to the corresponding author. The mRNA sequencing dataset supporting the conclusions of this article is available in the Sequencing Read Archive (SRA) database with the BioProject accession number PRJNA1133000 (https://www.ncbi.nlm.nih.gov/bioproject/PRJNA1133000) and the DataverseNO data archive with the persistent identifier 10.18710/BZOG3S.All materials used in this study are listed in Additional File 3.

## Abbreviations

HSC: Hepatic stellate cell
hPSC: Human pluripotent stem cell
scHSC: Stem cell-derived hepatic stellate cell
pHSC: Primary hepatic stellate cell
VA: Vitamin A
LD: Lipid droplet
TGF-β: Transforming growth factor beta
α-SMA: Alpha smooth muscle actin
PDGFR-β: Platelet-derived growth factor receptor beta
ECM: Extracellular matrix
ER: Endoplasmic reticulum
UPR: Unfolded protein response
ROL: Retinol
RAR: Retinoic acid receptor
RXR: Retinoid X receptor
REH: Retinyl ester hydrolase
PA: Palmitic acid
HT: Holotomography/ holotomographic
IF: Immunofluorescence
PCA: Principal component analysis
RT-qPCR: Real-time quantitative polymerase chain reaction
ELISA: Enzyme-linked immunosorbent assay
UV: Ultraviolet
OxPhos: Oxidative phosphorylation
GO: Gene ontology
