# Supplementary material for: The effects of TGF-β-induced activation and starvation of vitamin A and palmitic acid on human stem cell-derived hepatic stellate cells
Source: Stem Cell Res Ther. 2024 Jul 23;15:223. doi: 10.1186/s13287-024-03852-8 (PMC11267759; doi:10.1186/s13287-024-03852-8)
Supplement: Supplementary file 1 — Supplementary Material 1 [file 13287_2024_3852_MOESM1_ESM.docx]

**Supplementary Information**

**Document S1: Figures S1-S7**

**The Effects of TGF-β-induced Activation and Starvation of Vitamin A and Palmitic Acid on Human Stem Cell-Derived Hepatic Stellate Cells**

*Ingrid Wilhelmsen^1,2^, Thomas M. D. Combriat^2^, Andrea Dalmao-Fernandez^1,2,3^, Justyna Stokowiec^2^, Chencheng Wang^2,4^, Petter Angell Olsen^1,2^, Jonas Aakre Wik^1,2^,* *Yuliia Boichuk^2^, Aleksandra Aizenshtadt^1,2^, Stefan J. K. Krauss ^1,2^*

^1^ Department of Immunology and Transfusion Medicine, Oslo University Hospital, Oslo, Norway.

^2^ Hybrid Technology Hub - Centre of Excellence, Institute of Basic Medical Sciences, University of Oslo, Oslo, Norway.

^3^ Section for Pharmacology and Pharmaceutical Biosciences, Department of Pharmacy, University of Oslo, Oslo, Norway

^4^ Department of Transplantation Medicine and Institute for Surgical Research, Oslo University Hospital, Oslo, Norway.

A. Aizenshtadt and S. J. K. Krauss share senior authorship.

**Supplementary figure legends**

**Figure S1. (Related to Figure 1). Characterization of scHSCs.**

1. Gene expression of the pluripotency marker *OCT4* and HSC markers (*PDGFR-β*, *NCAM1*, *DESMIN*, *ALCAM*, *LRAT*, and *ACTA2*) comparing scHSCs to hPSCs and pHSCs. Relative expression was normalized to pHSCs. PSCs: n = 4 cell lines, N ≥ 2; scHCSs: n = 4 cell lines, N = 3; pHSCs: n = 2 donors.
2. Representative immunofluorescence confocal images of PDGFR-β, Vimentin, and NCAM1 in scHSCs and pHSCs. Scale bars: 40 µm.
3. Accumulation of lipids as measured by holotomographic (HT) imaging throughout the 12-day scHSC differentiation protocol. Retinol (ROL) and palmitic acid (PA) treatment was started on day 6. Treatment was not initiated in the “No ROL & PA” group, illustrating that ROL and PA facilitate lipid formation. Data from 4 independent experiments with n $\geq$ 3 cell lines were used, 268 ≥ N ≥ 16 for “scHSC differentiation”, and 52 ≥ N ≥ 36 for “No ROL & PA”.
4. Representative histograms of flow cytometry analysis showing PDGFR-β positive populations in all scHSC cell lines, pHSCs, and treatments. Gating was performed on samples stained only with secondary antibody (sAB) of pluripotent stem cells (PSC) for each cell line and pHSCs. Numbers indicate the percentage of the cell populations within the gate. St.: Starvation. scHSCs: n = 4 cell lines, N = 3 technical replicates (except scHSC_2, where N = 1); pHSCs: n = 1 donor, N = 3 technical replicates.µ

**Figure S2. (Related to Figure 1). Differential gene expression and activation of scHSCs and protein release in pHSCs.**

1. Total differentially expressed gene (DEG) count, showing all DEGs and DEGs up- and downregulated. n = 4 cell lines, N = 3 technical replicates.
2. Venn diagrams showing the number of genes expressed exclusively and commonly in each treatment group. n = 4 cell lines, N = 3 technical replicates.
3. Relative expression of HSC activation-related genes *ACTA2*, *COL1α1*, *PDGFR-β*, and *SMAD7* as measured by RT-qPCR. The samples are normalized to “Control”. n = 4 cell lines, N = 3 technical replicates.
4. Heat maps of genes related to the Wnt and Hippo signaling pathways. The displayed genes were detected through Kyoto Encyclopedia of Genes and Genomes (KEGG) enrichment analysis comparing the “Starvation + TGF-β” group to the “Control” group. The scale represents the z-score. n = 4 cell lines, N = 3 technical replicates.
5. Heat map of genes involved in the TGF-β/ Smad2/3 signaling pathway. The scale represents the z-score. R-SMADs: Receptor-regulated SMADs. *SMAD4 is not itself an R-SMAD, but is a mediator of R-SMAD signaling. n = 4 cell lines, N = 3 technical replicates.
6. Secretion levels of cytokines released by pHSCs in culture media after 24 hours of incubation, displayed as the fold difference compared to “Control”. n = 1 donor, N = 4 technical replicates. *p-value ≤ 0.05.

**Figure S3. (Related to Figure 2). VA-and LD-related genes and VA autofluorescence in scHSCs.**

1. Relative expression of HSC VA- and LD-related genes *LRAT*, *RBP1*, *RBP4*, and *PLIN2* as measured by RT-qPCR. The samples are normalized to “Control”. n = 4 cell lines, N = 3 technical replicates.
2. Representative bright-field and UV images of scHSC_4 and pHSCs, in the “Control” condition after 24h of incubation. Scale bars: 100 µm.
3. Representative histograms of flow cytometry analysis showing autofluorescence produced by VA in all scHSC cell lines, pHSCs, and treatments. Gating was performed on samples of pluripotent stem cells (PSC) for each cell line. St.: Starvation. scHSCs: n = 4 cell lines, N = 3 technical replicates (except scHSC_2, where N = 1); pHSCs: n = 1 donor, N = 3 technical replicates.

**Figure S4. (Related to Figure 2). Confocal raman spectroscopy of scHSCs and holotomographic (HT) imaging of scHSCs and pHSCs.**

1. Scatter plot of the vitamin A (VA) signal versus the lipids signal, both computed as the integral of the Raman signal in their respective windows, for every pixel of the imaged fields. The colors depicts the local density of scatters, from black to yellow, as computed by a gaussian-kernel density estimate, highlighting the correlation between the two compounds. n = 4 cell lines, N $\geq$ 3 independent fields imaged.
2. VA- and lipid detection in scHSCs as measured by Raman spectroscopy. n = 4 cell lines, N ≥ 3 independent fields imaged.
3. Characterization of intracellular lipid droplets in scHSCs and pHSCs for 7 days as detected from 3D live-cell HT images. scHSCs: n = 4 cell lines, N = 4 independent fields imaged. pHSCs: n = 2 donors (except for the “Starvation” group, where n = 1 donor), N ≥ 2 independent fields imaged. Error bars show the standard deviation.

**Figure S5. (Related to Figure 3). Seahorse analysis of scHSCs.**

1. Oxygen consumption rate (OCR) and extracellular acidification rate (ECAR) of scHSCs in the presence and absence of the glycolysis inhibitor 2-Deoxy-D-Glucose (2-DG). The values were normalized to the basal respiration of the “control” condition. Horizontal dotted lines indicate the baseline set to 100 %. Error bars show the standard error of the mean (SEM). Olig: Oligomycin, FCCP: Carbonyl cyanide 4-(trifluoromethoxy)phenylhydrazone, Rot: Rotenone, AA: Antimycin A. n = 4 cell lines, N $\geq$ 2 technical replicates.

**Figure S6. (Related to Figure 3). Glucose and fatty acid metabolism in scHSCs and pHSCs.**

1. Cell-associated glucose and oleic acid and fractional oxidation (oxidized substrate divided by uptake) of glucose and oleic acid in scHSCs after 48 hours of treatment. n = 4 cell lines, N = 4 technical replicates.
2. Glucose and oleic acid metabolism in scHSCs and pHSCs. scHSCs: Three independent experiments with n = 4 cell lines per experiment, N ≥ 4 technical replicates. pHSCs: n = 1 donor with N = 4 technical replicates.

**Figure S7. (Related to Figure 3). Lactate production in pHSCs and mitochondrial network analysis of scHSCs.**

1. Lactate in culture media and cell lysates of pHSCs after 48 hours of treatment. n = 1 donor, N = 4 technical replicates.
2. Mean degree of the mitochondrial network of scHSCs. The mean degree is computed as the average number of nearest neighbors for every pixel that is part of the skeleton. n = 4 cell lines, N = 5 independent fields imaged. The data was cleaned for outliers using ROUT, Q = 1 %.

**S1**


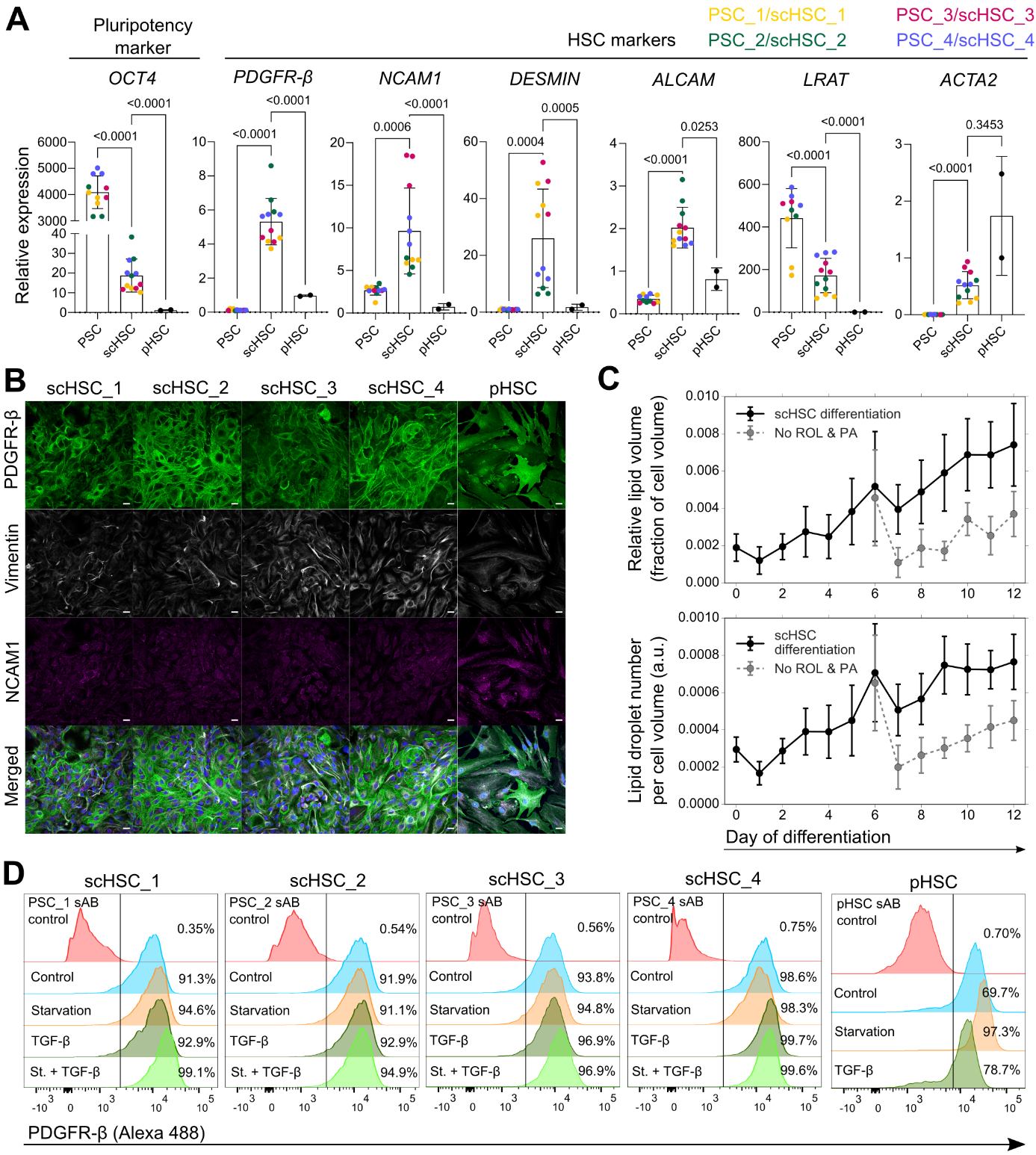


**S2**


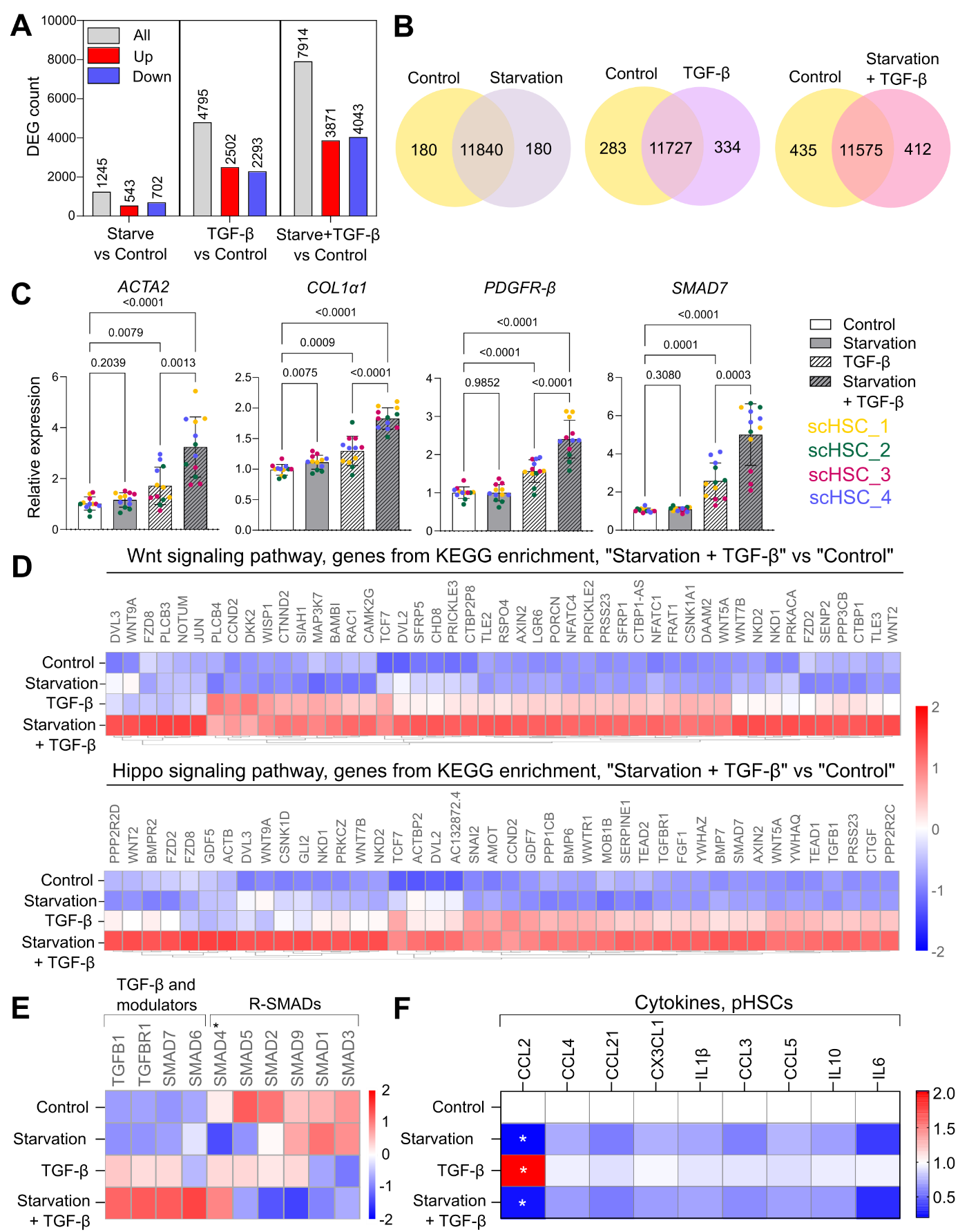


**S3**


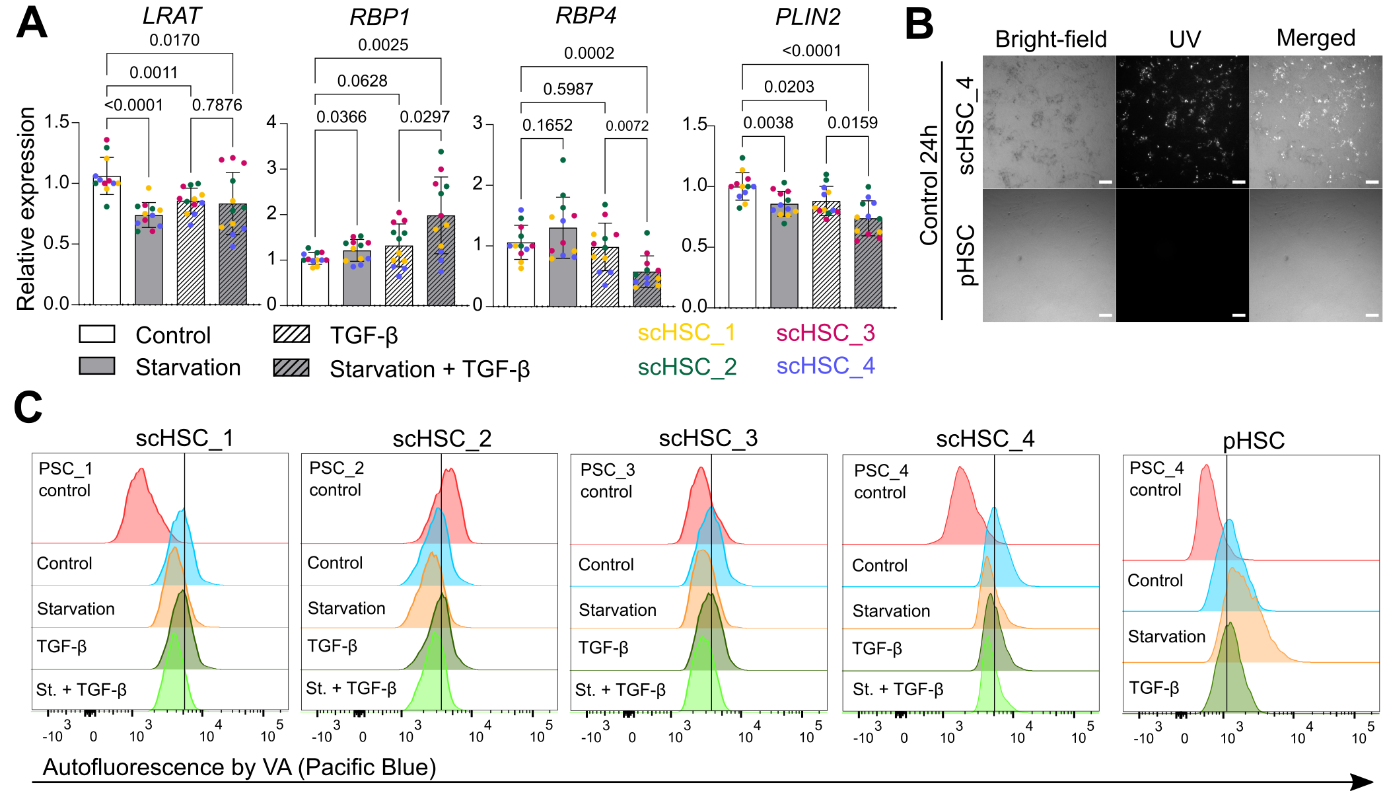


**S4**


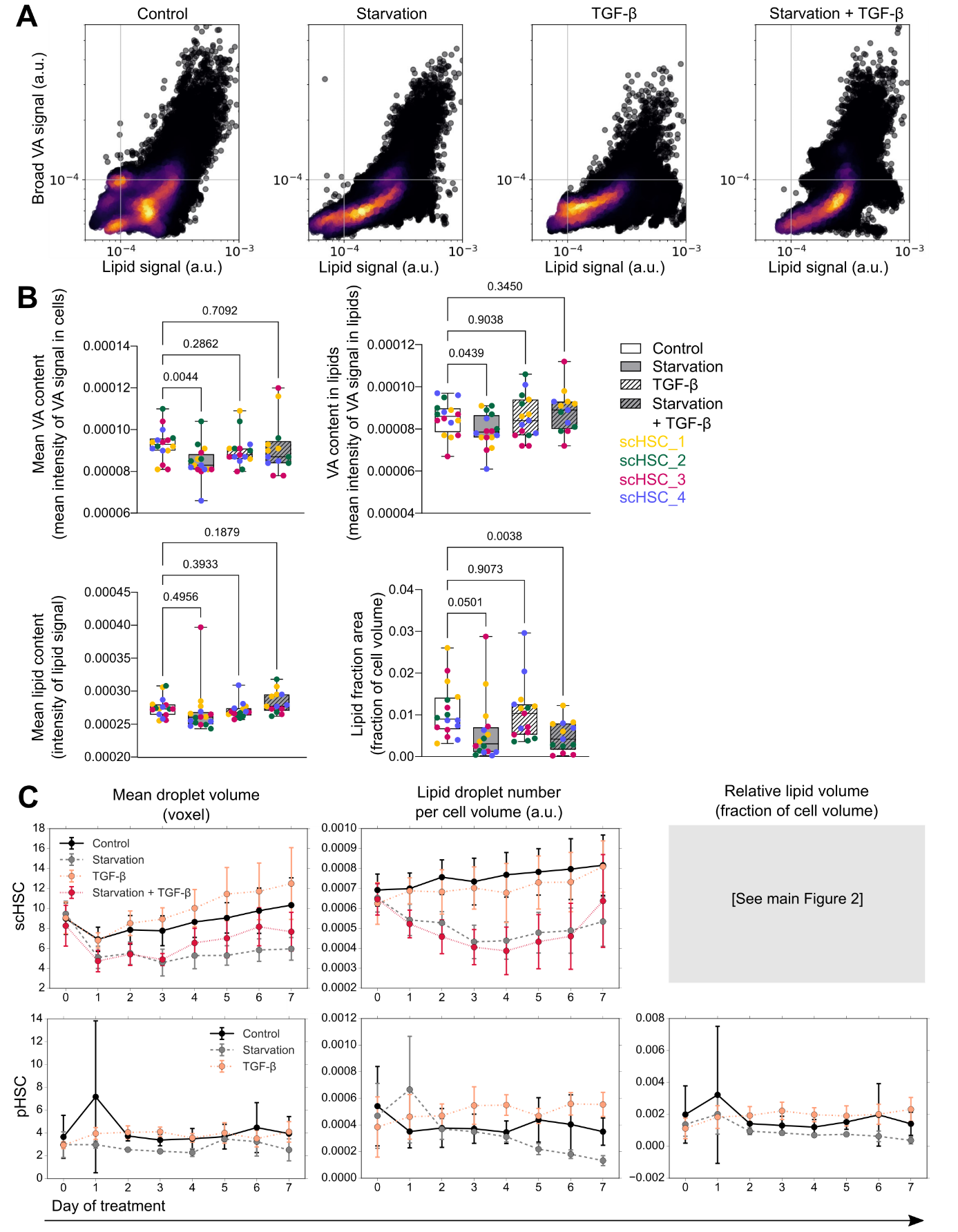


**S5**


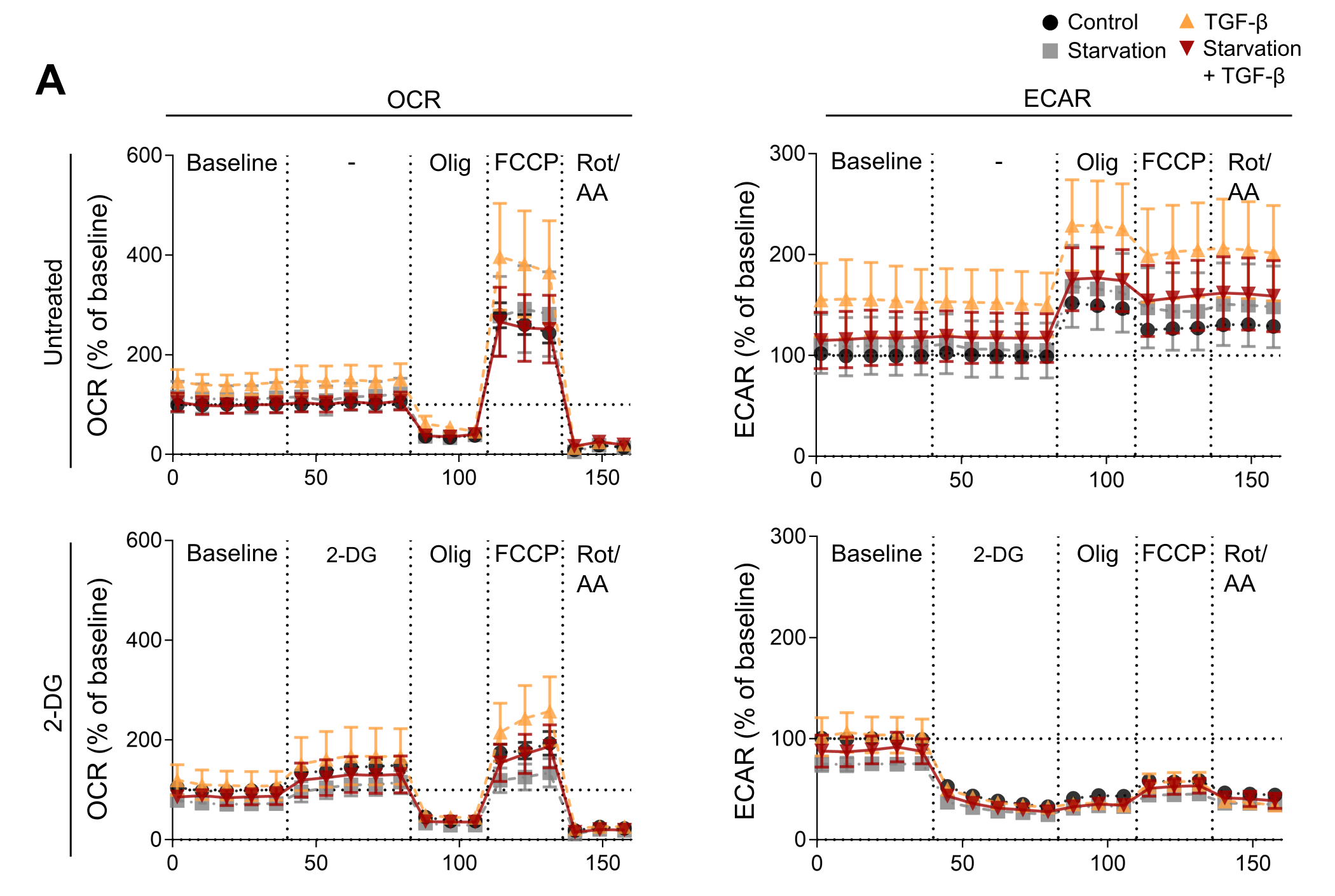


**S6**


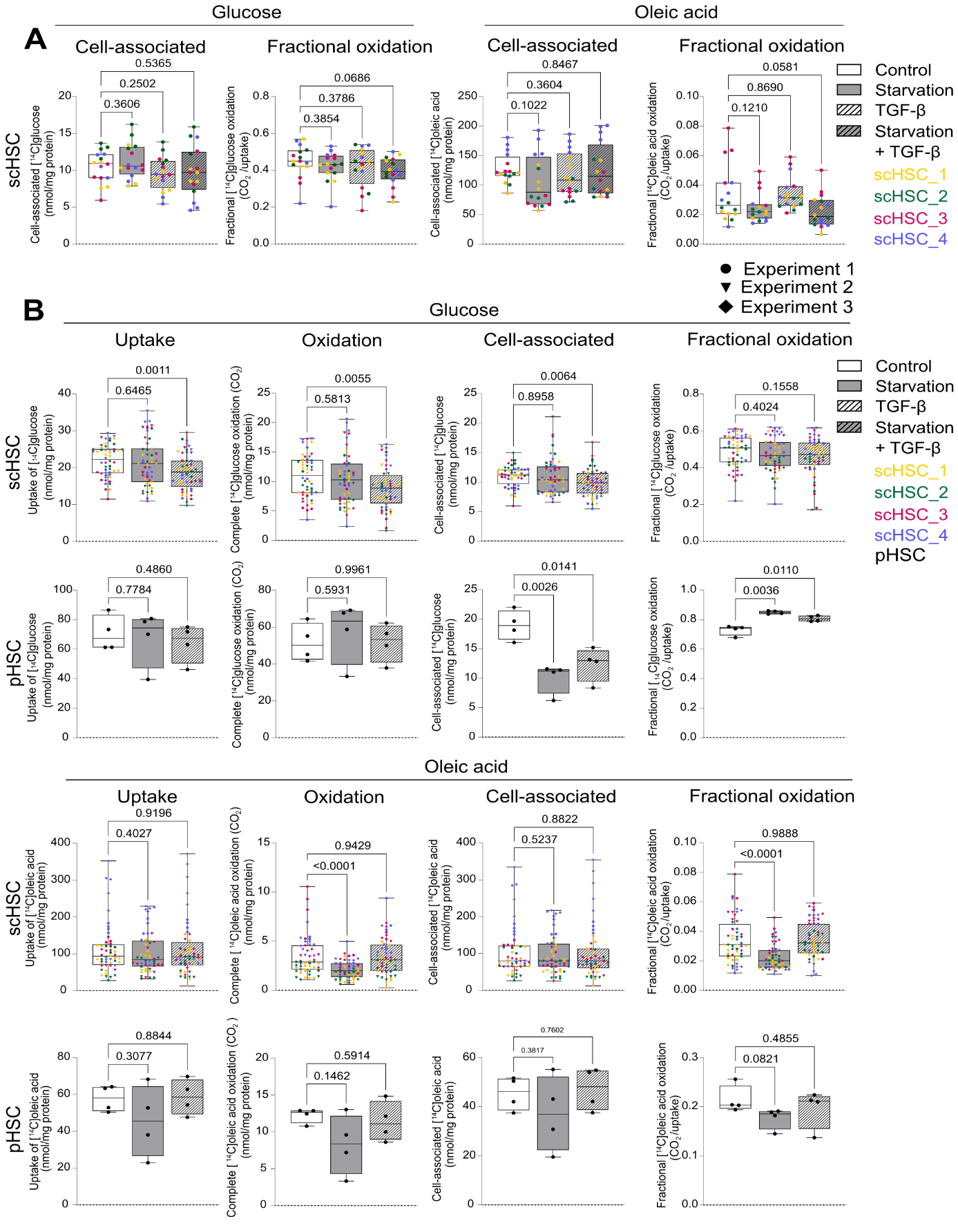


**S7**

**
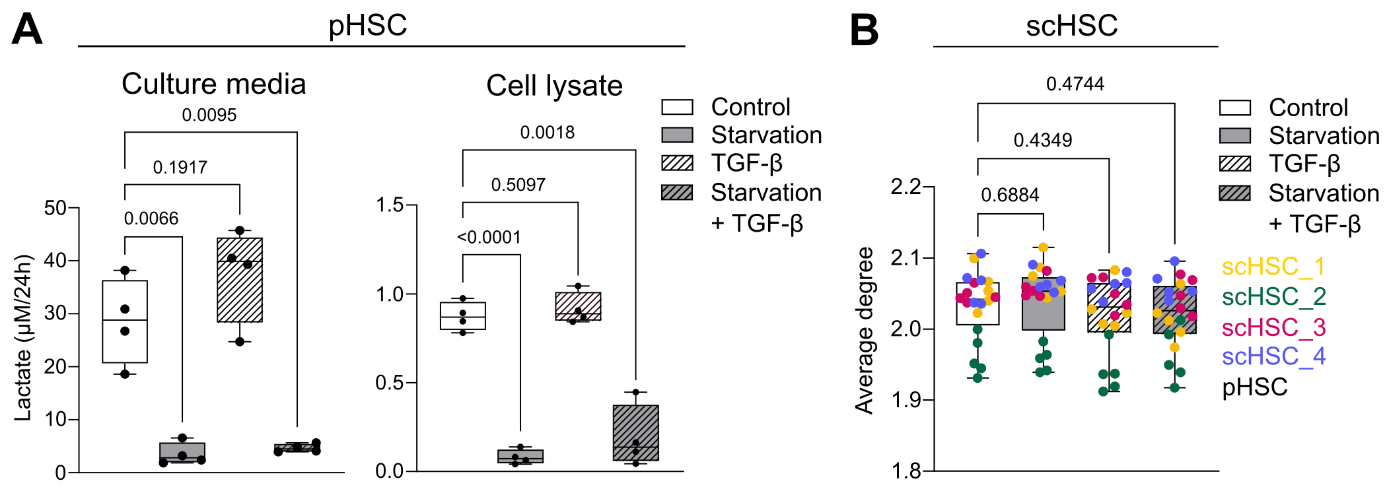
**
